# Reproductive Performance of the Alpine Plant Species *Ranunculus kuepferi* in a Climatic Elevation Gradient: Apomictic Tetraploids Do Not Show a General Fitness Advantage over Sexual Diploids

**DOI:** 10.3390/life14091202

**Published:** 2024-09-22

**Authors:** Ursula Ladinig, Elvira Hörandl, Simone Klatt, Johanna Wagner

**Affiliations:** 1 Department of Botany, Functional Plant Biology, University of Innsbruck, Sternwartestrasse 15, A-6020 Innsbruck, Austria; 2 Department of Systematics, Biodiversity and Evolution of Plants, Albrecht-von-Haller Institute for Plant Sciences, University of Goettingen, Untere Karspuele 2, D-37073 Goettingen, Germany; elvira.hoerandl@biologie.uni-goettingen.de (E.H.);; 3 Central Administration, University of Goettingen, Humboldtallee 15, D-37073 Goettingen, Germany

**Keywords:** apomixis, anthesis, flower development, geographical parthenogenesis, pollen tubes, polyploidy, reproductive success, reproductive mode, seed set

## Abstract

Previous studies on the mountain plant *Ranunculus kuepferi* concluded that apomictic self-compatible tetraploids have experienced a niche shift toward a colder climate during the Holocene, which suggests a fitness advantage over the sexual, self-sterile diploid parents under cold and stressful high-mountain conditions. However, there is still a lack of information on whether reproductive development would be advantageous for tetraploids. Here, we report on microsporogenesis, megagametogenesis, the dynamics of flower and seed development, and the consequences for reproductive success in a common garden experiment along a 1000 m climatic elevation gradient and in natural populations. Flower buds were initiated in the year preceding anthesis and passed winter in a pre-meiotic stage. Flower morphology differed in the known cytotype-specific way in that tetraploid flowers produced about twice as many carpels and fewer petals, stamens, and pollen grains than diploid flowers. Tetraploids developed precociously aposporous embryo sacs and showed a high rate of developmental disturbances. Sexual seed formation prevailed in diploids and pseudogamous apomixis in tetraploids. Along the elevation gradient, stigma pollen load, pollen performance, and seed output decreased. Combinations of reproductive traits, namely, bypass of meiosis irregularities and uniparental reproduction, might have promoted the vast expansion of apomictic *R. kuepferi* lines across the European Alps.

## 1. Introduction

Apomixis, the asexual mode of seed production, occurs in more than 290 plant genera within the major clades of angiosperms, which represents around 2.3% of plant genera up to date [1,2,3]. One possible reason for this relatively rare occurrence might be the complex evolution of apomixis in natural systems [4]. Since apomicts are mainly polyploids, polyploidization is one of the basic prerequisites to establish apomixis [5,6], which has been confirmed for a number of apomictic species in molecular studies ([4] for review). In most taxa, polyploidization follows interspecific hybridization (allopolyploidy); in some taxa, it follows intraspecific crossing (autopolyploidy) between sexual progenitors [5,7,8]. Sexual polyploids are widespread within angiosperms [9] and can be formed rapidly by unreduced gametes, either via a one-step formation or triploid bridge [10,11]. Besides genetic factors, adverse environmental conditions (reviewed in [12,13]), such as low temperatures [14,15,16], can also trigger the formation of unreduced male and female gametes in the sexuals. Thereby, unreduced embryo sacs can be formed in parallel or alternatively to sexual ones; equally, pollen meiosis may be imperfect or does not take place at all. Unreduced gamete formation can lead to the formation of apomictic seeds in diploids and is the first step toward gametophytic apomixis in plants. The shift from the sexual to a stable apomictic mode is much more complex and requires the simultaneous establishment of various genetic and/or epigenetic regulatory mechanisms [17,18,19]. Disorders in the sexual system and shifts in the timing of developmental steps caused by hybridization and/or polyploidization seem to favor the shift to apomixis [5,20,21,22]. However, polyploidization is not an absolute prerequisite for the apomictic pathway, as shown for the diploid, apomictic Brassicaceae *Boechera* [23], and synthetic diploid hybrids in the *Ranunculus auricomus* complex [24,25,26].

In taxa with established apomixis, genetic and/or epigenetic changes alter the reproductive pathway to make apomixis functional. Three independent processes must act together to produce functional apomictic seeds: (1) the development of an unreduced embryo sac bypassing meiosis via diplospory or apospory; (2) the egg cell develops an embryo without fertilization via parthenogenesis; and (3) overcoming imprinting barriers to form a functional endosperm [4]. Endosperm development is crucial for embryo development and, in the case of pseudogamy, is initiated by the fertilization of the polar nuclei or the secondary embryo sac nucleus by one or two reduced or unreduced sperm nuclei [27,28], giving rise to a biparental endosperm and a maternal embryo. In some taxa (e.g., Asteraceae), endosperm forms autonomously without fertilization; consequently, all seed tissues are purely maternal [28].

Most apomicts maintain facultative sexuality to a variable degree, whereby environmental factors such as temperature [16], photoperiod [29,30], drought [31], starvation, and oxidative stress [32] can additionally influence the reproductive mode. Sexual and apomictic embryo sacs can develop in parallel on the same mother plant in the same or different flowers and within a flower, in the same or different ovules [2,7]. The apomictic way of seed production may be advantageous when establishing and maintaining populations under demanding environmental conditions. This becomes evident in the fact that apomictic taxa often show latitudinally and elevationally larger distribution ranges than their sexual progenitors and tend to colonize previously glaciated areas more frequently—a phenomenon known as “Geographical Parthenogenesis” (GP) [33,34,35,36,37,38]. Polyploidization frequently causes a breakdown of self-incompatibility [39,40]. Consequently, apomictic plants are mostly self-fertile in contrast to their sexual (non-polyploid) relatives [7,41,42]. Uniparental reproduction provides reproductive assurance when pollinators are uncertain or missing due to strongly fluctuating weather conditions, as is the case in cold climates [43]. Furthermore, apomixis may reduce the time span for developmental pathways by precocious development of the embryo sac [28,44], which could be an advantage for apomicts in short vegetation periods. This is because parthenogenetic development of unreduced egg cells can start independently from pollination and result in precocious embryo formation. It is likewise important that single apomictic individuals arising from single seeds after long-distance dispersal, e.g., by animals, can found new populations (“Baker’s law” [45]), which would not succeed in a single sexual outcrossing individual.

Generally, polyploidy might give apomicts a fitness advantage over the diploid parents in stressful habitats and is, therefore, supposed to be one of the main drivers for niche differentiation [34,35,46,47]. The combination of genetic information from different ancestors and/or the multiplication of alleles might provide a higher developmental plasticity and stress tolerance compared with the diploid parents [48]. Though non-apomictic polyploids do not necessarily have wider distribution ranges than diploid relatives [49], the general tendency of polyploids to be associated with colder climates [50] could also play into the development of GP.

Apomictic reproduction may also have disadvantages, particularly in evolutionary younger taxa where the apomictic developmental pathway has not yet stabilized [25,51]. Pseudogamous apomicts often show a lower seed set than their sexual relatives, which, due to developmental disturbances, show reduced pollen fertility and often a high percentage of sterile ovules and aborted seeds [7,42,51,52].

The reconstruction of the evolution of GP in different plant taxa is based mainly on molecular, cytological, ecological, and morphological data collected in extant sexual and apomictic populations and on theoretical modeling with these data (e.g., [16,42,46,53,54,55,56]). However, there is a lack of studies comparing the reproductive development of cytotypes under the same growing conditions in a natural climatic gradient. Such common garden experiments on the example of appropriate model species could indicate which of the hypothetic cytotype-specific features that might have led to GP after the retreat of the Pleistocene glaciers are most relevant.

*Ranunculus kuepferi* is an alpine perennial herb appropriate for studying acclimation to cold conditions [57]. The species shows a distinct GP pattern within the European Alps. Diploid sexuals are restricted to their glacial refugia in the Southwestern Alps, whereas tetraploid apomicts colonize almost the whole Alpine Arc, the Northern Apennines, and the Corsica mountains [51,53,58]. In the Alps, apomicts colonized higher elevations (1700–2800 m a.s.l.) than sexual diploids (1300–2500 m a.s.l.) [46,51] and conducted a niche shift of apomicts toward colder conditions and more acid soils [46,55,56].

In a multiple common garden study on diploid and tetraploid *Ranunculus kuepferi* plants along a 1000 m climatic elevation gradient, we assessed various response parameters concerning growth and reproduction during four consecutive years following transplantation. Single examinations were also carried out in one natural diploid and one tetraploid population. In a parallel report [59], we concentrate on vegetative growth, frost tolerance, flowering frequency, and reproductive success and state that sexuals performed equally or even better than apomicts also at the highest site. However, tetraploids showed single features that might be advantageous for survival in a cold, stochastic climate: (1) Freezing tolerance of leaves increased with elevation in both cytotypes but tended to be higher in tetraploids than in diploids. (2) Leaf mass and specific leaf area were significantly higher at the highest site than in diploids, increasing the photosynthetic active tissue per unit leaf area, which (3) may cause tetraploids to invest more into rhizome storage. And (4), though seed output was minimal, tetraploids showed a greater tendency to establish seed banks than diploids.

In the present report, we focus on the ontogeny and developmental dynamics of apomictic and sexual structures, depending on elevation. We investigated the whole reproductive cycle from floral initiation through flower development, anthesis, and seed development to seed maturity. Embryological details in ovule, pollen, and seed development and the proportion of functional and dysfunctional structures should show to what extent sexual and apomictic ontogenesis are influenced by climatic site conditions. Additionally, we tested whether elevation has an impact on the reproductive mode in seeds of both cytotypes via flow cytometric seed screening.

## 2. Materials and Methods

### 2.1. Characteristics of the Study Plant

*Ranunculus kuepferi* Greuter & Burdet (Ranunculaceae) is a perennial clonally growing herbaceous plant. The shoot system is a belowground sympodial rhizome consisting of a cluster of individually rooted ramets. Each ramet forms a variable number of grass-like leaves, forming a basal rosette (Figure S1a). After a vegetative phase, the apical meristem of a ramet becomes floral, giving rise to a uni- or few-flowered inflorescence. From axillary buds, renewal shoots develop and, after rooting, disintegrate into daughter ramets. Flowers of both sexual diploid (2*x*) and apomictic tetraploid (4*x*) plants are hermaphroditic (Figure S1b,c). At the study sites in natural populations (2*x*: Colle della Lombarda, 4*x*: Kaun Valley, Table S1), sexual flowers consisted of 5 sepals, 5 petals, 32–85 stamens, and 3–30 free uniovular carpels in spiral arrangement (Figure S1b). In apomictic flowers, sepal and petal numbers were irregular (0–5); the number of stamens was lower (11–37), and the number of carpels was significantly (*p* < 0.001, *t*-test) higher (up to 100) than in sexuals (Figure S1c). Each flower gives rise to an aggregate fruit consisting of a variable number of fruitlets (=achenes). Diploid sexuals exhibit stylar self-incompatibility, whereas tetraploid apomicts are aposporous, pseudogamous (with rare autonomous apomixis), and self-compatible [42,46,57]. Thus, the sexuals—in contrast to apomicts—largely depend on pollinators and mating partners to set seeds. Nevertheless, sexual *R. kuepferi* plants generally have a higher seed set than apomicts which, due to developmental disturbances, show reduced pollen fertility and often a high percentage of sterile ovules and aborted seeds [16,42,46,51,60].

### 2.2. Origin of the Plant Material and Experimental Design

#### 2.2.1. Container Plots along the Elevation Transect

To test whether there are differences in the fitness performance between diploid sexuals and tetraploid apomicts depending on the site climate, individuals of both cytotypes of different proveniences (Table S1) were transplanted along a 1000 m elevation gradient in the Stubai Alps (Austrian Central Alps, 46°59′ N, 11°06′ E). In the second half of August 2014, experimental plots were installed at a subalpine site (1770 m a.s.l, hereinafter referred to as P1800), a lower alpine site (2290 m a.s.l., P2300), a higher alpine site (2600 m a.s.l., P2600), and a subnival site (2830 m a.s.l., P2800) (Figure S2a–d). The subalpine and alpine plots were situated within dense grasslands, which are extensively grazed by cows (subalpine site) and sheep (alpine site). The higher alpine plots were set up on a flat moraine ridge, and the subnival plots on a flat, rocky plateau. The vascular plant cover was sparse at both upper sites. To eliminate the influence of competition with the natural vegetation and different soils at the different sites and to focus on the effect of the different climatic site conditions, container plots were established. Plastic grid baskets (26 × 28 cm) were lined with garden fleece, filled with lime-free standard alpine soil, and sunk into the ground. Two directly adjacent containers formed one plot in which 18 single ramets from different individuals of different proveniences per cytotype were planted in a random arrangement (Figure S3a). To follow the development of each single individual, the root zone was delimited with a plastic mesh (Figure S3b). Three plots per cytotype were established per site; this gave 54 individuals per elevation and ploidy (Figure S3c) and a total of 432 transplanted individuals along the transect. Plots were protected from damage caused by grazing animals and birds with wire baskets (Figure S3d).

Study plants were collected in uniform diploid and tetraploid populations. Cytotype and genetic structure of the populations are known from earlier investigations [42,51]. The diploid individuals originated from two populations in the Western Alps (Table S1). Within the tetraploid plots, about half of the individuals originated from populations in the Western Alps and half from the Eastern Alps. Because of the clonal growth of this species, individuals were not clearly definable. To obtain different individuals as far as possible, sampling took place at distances of at least 5 m. Ramets that had flowered were excavated with the root balls at the end of the fruiting period when the aboveground organs started to senesce. Samples were transported in cooler bags to the Institute of Botany in Innsbruck and kept in a cooling chamber at 5–10 °C in dim light during the daytime until further processing. Plants were dissected into ramets; the largest ones were planted into the containers, which were then transferred to the sites along the transect. Main investigations in the plots were carried out in the growing seasons of 2015–2017, with single additional measurements taken in 2018 (Table S2).

#### 2.2.2. Study Sites in Natural Populations (NAT)

Diploids were studied in the extended stands around the Colle della Lombarda in the Western Alps (Table S1, population 1) at three different sites between 2260 and 2350 m a.s.l. in the summer of 2014 (Figure S4). Tetraploids were investigated in the upper Kaun Valley in the Eastern Alps (Table S1, population 6) at three different sites between 2470 and 2580 m a.s.l. in the growing season of 2014 (Figure S5).

#### 2.2.3. Climate Measurements

Microenvironmental temperatures were recorded at all sites at hourly intervals throughout the investigation period using small data loggers (Tidbit, Onset, Bourne, MA, USA). Temperature loggers were placed at the soil surface within each plot and covered by a white plastic boot to avoid solar radiation errors. Periods with snow cover and the exact melting dates could be derived from the logger data. From the hourly temperature values in the plots, daily plot means, from these daily site means, and finally, total means for the growing period of *R. kuepferi* (snowmelt until seed maturity) were calculated.

### 2.3. Timing of Flower Development and Flowering Phenology

**Figure 1:**
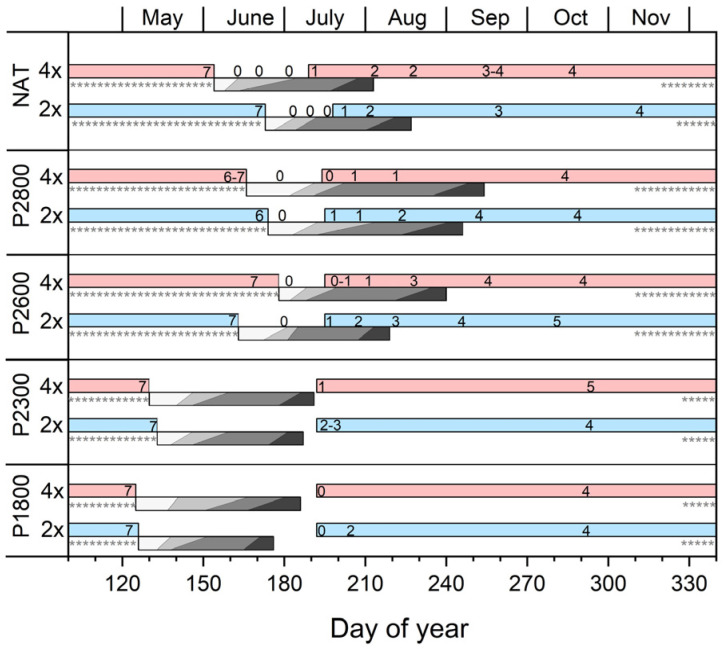
Floral development and reproductive phenology in the natural stands (NAT) and the plots along the elevation transect (P1800–P2800) in *R. kuepferi* plants. Progress of floral development in diploids (2*x*, blue bars) and tetraploids (4*x*, red bars) is shown in the year of floral initiation (bars at the right) and before snowmelt in the year of flowering (bars at the left). Numbers in the bars specify the mean developmental stage of the shoot apices at the different sampling dates from 0 (shoot apex still vegetative) and 1 (early floral apex) to 7 (postmeotic prefloral stage); for staging, see Figure 2. Grey-edged bars show the sequence of reproductive phenophases: prefloration period (white); anthesis (light grey); fruit/seed development (dark grey); and fruit/seed maturity (black). Asterisks indicate winter snow cover.

To investigate the dynamics of floral development, whole ramets were sampled between snowmelt in spring and the end of the growing season (for the sampling dates, see Figure 1 and for the number of collected ramets at the different sites, see Table S2—Flower development) and fixed in FPA50 (formalin, propionic acid, 50% ethanol, 5:5:90). Since for this purpose, individuals had to be dug up from the plots, sample collection took place in the last research year. Whole shoot tips in the early stages of floral development and anthers and ovules from flower buds in the prefloration phase were excised under a binocular microscope and examined in a clearing solution (lactic acid 85%, chloral hydrate, clover oil, xylol, 2:2:2:1; based on [61]) under a microscope with differential interference contrast optics, DIC (Olympus BX50, Tokyo, Japan). The following developmental stages were distinguished: Stage 1: flower initiation (the apex enlarges and becomes dome-shaped; sepal primordia begin to emerge); Stage 2: sepal primordia are clearly visible, and first stamen primordia appear; Stage 3: all stamen primordia are visible, and sepals begin to elongate; Stage 4 (first carpel primordia appear) and Stage 5 (sepal, petal, stamen, and the majority of all carpel primordia have formed; stamens are still not differentiated into anthers and filaments; ovules are not yet visible) are the overwintering stages of the flower buds; Stage 6: meiosis/aposporous initial; Stage 7: prefloral (onset of embryo sac and pollen development until immediately before the beginning of anthesis).

The sequence of reproductive phenophases was documented between snowmelt and seed maturity both in the transect and at the natural sites. To always observe the same individuals at each of the three natural sites, a total of 76 (25, 25, 26) diploid and 70 (30, 29, 11) tetraploid individuals were marked with colored plastic pickers as soon as they emerged from the winter snow at the edge of snowfields (Figure S6a,b); phenological records were made at 2–4 d intervals. In the plots along the transect, the phenological status of all flowering individuals was assessed at 2–4(6) d intervals. At each census date, the numbers of flower buds, open flowers, developing fruits, and mature fruits were counted, and the percentage shares were calculated.

### 2.4. Duration of Anthesis and Seed Development on a Single Flower Basis

At the natural sites and in the transect plots, flowers of different individuals were labeled with small colored plastic rings at the beginning of anthesis (*n* flowers per cytotype = 150 at the natural sites and, depending on availability, 17–32 diploid flowers and 15–29 tetraploid flowers at each elevation in the transect plots). Rings were color-coded by the day of labeling. At the natural sites, 10 labeled, open-pollinated flowers or fruits from different individuals of each cytotype were sampled from the date of labeling until fruit maturity at 1–2 d intervals during anthesis and histogenesis and at 3–4 d intervals during seed maturation. In the plots—because of the limited number of flowers—we did not sample whole flowers but sampled 10–15 single carpels from at least 5 labeled, open-pollinated flowers from different individuals per cytotype and elevation at the same sampling intervals, as indicated for the natural sites. The withdrawal of the carpels was performed carefully with fine forceps without injuring the receptacle or other parts of the flower. Samples were fixed in FPA50 and stored until further processing. Ovules and developing seeds were examined in the clearing solution according to [61] using DIC microscopy, as described in Section 2.3. The length of the carpel, seed (longest axis between chalaza and micropyle), endosperm, and embryo were measured with image analysis software (Olympus, Cell^D, ver 3.1). From the embryological state at each sampling date, the duration of the following developmental phases was determined: prefertilization phase (onset of anthesis until fertilization); histogenesis (postfertilization until endosperm fully cellular); and seed maturation (end of histogenesis until seed dispersal).

### 2.5. Male and Female Performance

#### 2.5.1. Anthers and Pollen

The number of anthers per flower was determined in 47–62 diploid and 40–70 tetraploid flowers at the different sites. To determine pollen grain number, pollen viability, and pollen grain size, a number of flowers from different individuals per study site and cytotype were collected right before anther dehiscence and fixed in FPA50. From this stock, 10 flowers per study site and cytotype were used to determine pollen grain number and pollen viability following [62]: 5 anthers from each flower were squashed in a 0.5 mL Eppendorf vial in a mixture of 150 µL 1% acetocarmine dissolved in 45% acetic acid and 350 µL 50% ethanol with 0.5% Triton X-100. The pollen suspension was sonicated for 3 min to separate pollen grains from the anther and each other. Immediately after sonication, a small portion of the pollen suspension was injected into a Fuchs–Rosenthal dual chamber with a microliter pipette. In each of the two counting grids, the number of pollen grains was counted in 5 large squares of 1 mm^2^ (i.e., 10 squares per pollen sample), distinguishing between viable and non-viable pollen grains. Criteria for viability were a regular shape and a bright pink staining of the cytoplasm and the nuclei with acetocarmine. Small, shrunken, and irregularly colored or colorless pollen grains were considered non-viable. The mean number of pollen grains per square volume (0.2 µL) was multiplied by the dilution factor. Division by 5 and multiplication by the number of anthers of the tested flower gave the number of pollen grains per anther and flower, respectively. The pollen grain size was measured in a separate approach on an average of 22 flowers from the above-mentioned stock: 5 anthers per flower were gently squashed in a drop of acetocarmine on a broad microscope slide, and the diameter of at least 60 vital appearing pollen grains was microscopically assessed at 40× objective magnification using an image analysis software (Olympus, Cell^D, ver 3.1). The same preparation method was used to determine pollen viability in a further year of investigation.

#### 2.5.2. Carpels and Ovules

The number of carpels per flower was counted under a dissecting microscope in 80 diploid flowers and 145 tetraploid flowers from the natural sites and in an average of 70 diploid flowers and 50 tetraploid flowers per elevation in the transect. To assess the proportion of intact ovules, between about 100 and 250 randomly collected carpels of at least 20 flowers per cytotype and study site were microscopically investigated in the clearing solution according to [61] using DIC microscopy, as described in Section 2.3. The number of ovules with well-developed, malformed, or missing embryo sacs was counted, and their proportion was calculated.

#### 2.5.3. Seed Set

About one week before seed maturity, all available aggregate fruits in the plots and 100 in each of the natural stands were individually bagged with transparent fine-mesh organza to prevent the loss of fruitlets (Figure S3e). Mature aggregate fruits were harvested and individually stored in Eppendorf tubes at 5 °C. Under a dissecting microscope, the number of intact and undeveloped (ovules not fertilized or aborted) seeds per aggregate fruit was counted, and the percentage of ovules that successfully developed into seeds was calculated. At each elevation in the transect, the total number of analyzable aggregate fruits during 3 study years was 29–109 in 2*x* plants and 42–71 in 4*x* plants. At the natural sites, 70 (2*x*) and 95 (4*x*) aggregate fruits from different individuals were analyzed.

#### 2.5.4. Pollen/Ovule Ratio

The total pollen/ovule ratio (P/O_total_) was calculated from the total number of pollen grains and the total number of ovules (intact and defective) in 10 terminal flowers from 10 different individuals per cytotype and study site. Additionally, the P/O ratio was calculated from the number of intact appearing pollen grains and the number of intact ovules (P/O_intact_).

#### 2.5.5. Stigma Pollen Load, Pollen Germination, and Pollen Tube Growth

Stigma pollen load was determined in *n* = (40)60–102 carpels of 8–15 open-pollinated flowers per plot site and 164 (2*x*) and 307 (4*x*) carpels of 27 and 23 flowers, respectively, at the natural sites. At the end of the anthesis, when petals had faded, and stigmas did not appear receptive any longer, 3–4 carpels from each flower were collected and immediately transferred into a drop of glycerol on a slide. The rest of the flowers were fixed in FPA50. In situ fixation directly on the microscopic slide ensured that no pollen grains were lost. In the first step, the total number of pollen grains per stigma was counted. Then, stigmas were rinsed in 50% ethanol and gently vortexed to remove non-adhering pollen grains. The remaining pollen grains were counted again, distinguishing between adhering but ungerminated grains, germinated grains, and grains that had grown pollen tubes into the style. Pollen tube growth in the style was examined by the fluorescence standard method with aniline blue. In total, 30–60 carpels were excised from the fixed flowers (see before), washed twice in distilled water for 15 min each, transferred in 8N NaOH solution in 2 mL Eppendorf tubes, heated in a block heater at 55 °C for 5 min, rinsed twice more in distilled water for 1 h each, transferred to a slide in a drop of 0.1% aniline blue in Sörensen phosphate buffer, pH 8, covered with a cover slip, and stained for at least 2 h at room temperature. Carpels were gently squashed and examined under a fluorescence microscope (Olympus BH2, Tokyo, Japan; excitation filter 405–435 nm). The number of pollen tubes reaching the lower part of the style was determined. For the number of analyzable carpels, see Table S2—Pollen tube growth.

### 2.6. Reproductive Mode

The reproductive mode (sexual or apomictic) was determined by flow cytometric seed screening (FCSS), which measures the quantity of the genome in the embryo and the endosperm [63]. For 8-nucleate *Polygonum*-type embryo sac development, sexual seeds typically show peaks for C2*x* (embryo) and C3*x* (endosperm) tissues, pseudogamous apomictic seeds for 4*x* (embryo), and 10 or 12*x* (endosperm). From the 100 achenes collected per cytotype and elevation, we analyzed a total of 389 (2*x*) and 253 (4*x*) intact seeds from 76 (2*x*) and 48 (4*x*) individuals in two different study years and pooled data over the years to reach a minimum of 50 seeds per cytotype and elevation. On average, 5.2 intact seeds per individual were analyzed, comparable to [46] (Table S3). Seed ploidy was determined according to [16]. In brief: Single seeds were ground with a Tissue Lyzer II (Quiagen, Hilden, Germany); nuclei were isolated in Otto buffer I, filtrated, and suspended in Otto buffer II containing DAPI for DNA staining. The nuclei suspension was measured in a flow cytometer (CyFlow Space, Partec GmbH, Münster, Germany) in the blue fluorescence channel (UV LED, wavelength 365 nm). The DNA content (ploidy) of the nuclei is proportional to the detected fluorescence intensity. A diploid *R. kuepferi* plant was used as the external reference to adjust the gain standard of the UV lamp [46]. Gaussian means of the peaks were analyzed with the software FloMax version 2.81 (Quantum Analysis GmbH, Münster, Germany), and peak indices (mean peak value of the endosperm divided by mean peak value of the embryo) were calculated. Typically, the peak index of sexual seeds is around 1.5 (embryo:endosperm = 2C*x*:3C*x* and 4C*x*:6C*x* for diploid and tetraploid plants, respectively). Peak indices above the threshold of 1.9 indicate asexually formed seeds with an unreduced egg cell developing parthenogenetically into an embryo (2C*x*) and the two unreduced polar nuclei (4C*x*) fertilized by either reduced (1C*x*) or unreduced (2C*x*) male sperm nuclei (pseudogamous endosperm, i.e., embryo:endosperm = 2C*x*:5C*x* or 6C*x* for diploids and 4C*x*:10C*x* or 12C*x* for tetraploids). Representative histograms for *R. kuepferi* are presented in [16,46]. In cases of undetectable small embryo peaks, we used either the ratio of the endosperm peak to leaf material of the mother plant or an internal standard with constant genome size (*Pisum sativum*) to determine the ploidy of the endosperm. Seeds with a ploidy shift in the embryo (so-called BIII hybrids resulting from fertilization of an unreduced egg cell, and a peak index of c. 1.7 (see [11,16]) were very rare and were excluded from statistical evaluation (Table S3).

### 2.7. Statistics

The number of investigated structures per parameter, site, and cytotype is given in Supplementary Data Table S2. Metric data (temperature, duration of prefloration and postfloration, pollen parameters, number of ovules, P/O ratio) were tested for normality and homogeneity of variance (Levene’s test). Statistical differences among sites within the same cytotype were tested with one-way ANOVA and Duncan post-hoc comparison (equal variances, equal number of cases) or Games–Howell post-hoc comparison (unequal variances and/or unequal number of cases). Statistical differences between cytotypes at the same site were tested by *t*-test for equal or unequal variance. Statistical differences between data on a nominal scale (reproductive mode) were tested by cross-tables and a chi-square test (Pearson). All tests were made with the statistical package SPSS ver. 29.0.2.0 (IBM, Armonk, NY, USA) at the critical level of significance α = 0.05.

## 3. Results

### 3.1. Climate and Vegetation Period

Due to the staggered melting dates along the transect, the total mean temperatures in the period between snowmelt and seed maturity of *R. kuepferi* plants did not decrease uniformly with elevation but were about the same up to P2600 (11–12 °C) and were only significantly lower (9.6 °C; *p* ≤ 0.001; one-way ANOVA, Games–Howell post-hoc comparison) at the subnival site P2800 (Table S4). Mean temperatures in the natural 2*x* population in the Western Alps were significantly higher among all sites (13.4 °C; *p* ≤ 0.034), whereas the mean temperature in the natural 4*x* population in the Eastern Alps was within the range measured at the highest plot site P2800 (9.9 °C). Temperature means did not significantly differ between the 2*x* and 4*x* plots at the same elevation; however, they significantly differed (*p* < 0.001, *t*-test) between the natural sites of both cytotypes.

### 3.2. Timing of Flower Development and Reproductive Phenology

**Figure 2:**
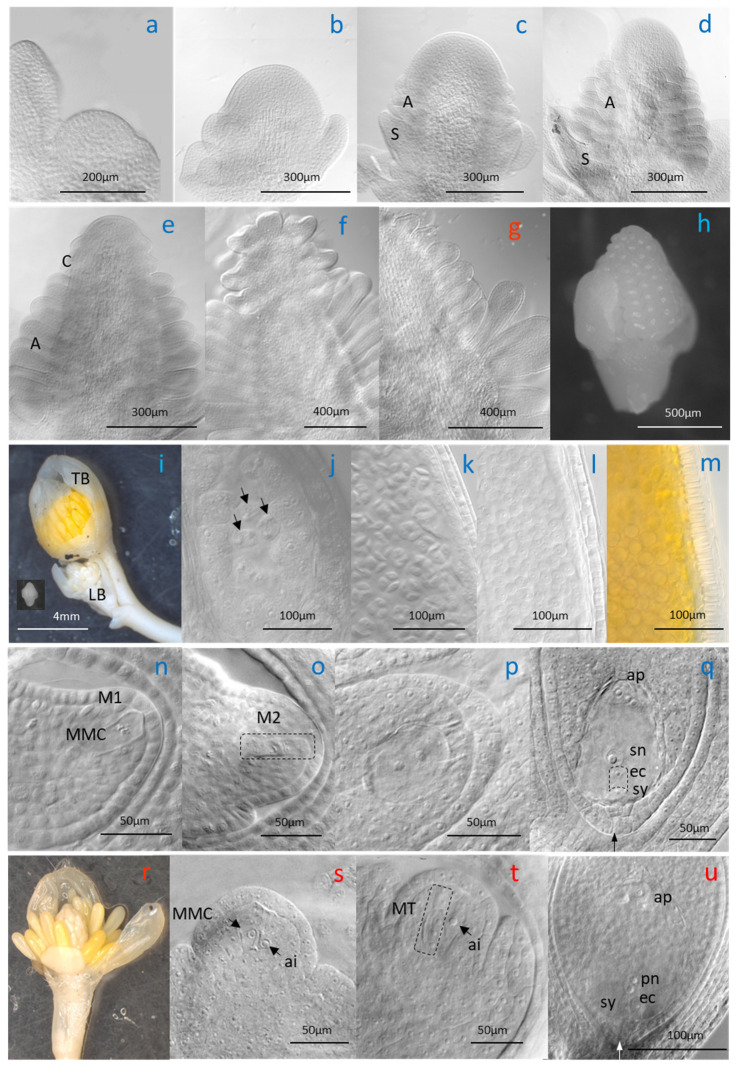
Stages of floral development in diploid (blue letters (**a**–**f**,**h**–**q**)) and tetraploid (red letters (**g**,**r**–**u**)) *R. kuepferi* plants. (**a**) Stage 0: vegetative shoot apex. (**b**) Stage 1: dome-shaped floral meristem. (**c**) Stage 2: sepal primordia (S) and first stamen primordia (A) appear. (**d**) Stage 3: all stamen primordia are visible; sepal primordia begin to elongate. (**e**) Stage 4: first carpel primordia (C) appear. (**f**) Stage 5: all carpel primordia are visible. (**g**) Stage 5: tetraploid plant. (**h**) Stage 6: diploid plant; flower bud before winter. (**i**) Stage 7: greatly enlarged terminal flower bud (TB) in a postmeiotic state at snowmelt in spring (large picture) compared to the pre-winter stage (small picture at the lower left); smaller lateral flower buds (LB) were still in a premeiotic state. (**j**–**l**) microsporogenesis in diploid plants**:** (**j**) meiosis (arrows point to microspore mother cells at telophase 1); (**k**) microspore tetrads; (**l**) microspores; (**m**) mature pollen grains. (**n**,**o**) megasporogenesis in diploid plants: (**n**) megaspore mother cell in metaphase of meiosis 1 (M1); (**o**) megaspore tetrad after meiosis 2 (M2). (**p**,**q**) megagametogenesis in diploid plants: (**p**) 2-celled and (**q**) 7-celled *Polygonum* type embryo sac with antipodes (ap), central cell with the secondary embryo sac nucleus (sn), egg cell (ec) and synergides (sy); (**r**) flower bud of tetraploid plant at snowmelt in spring; (**s**–**u**) tetraploid plant, ovules during development: (**s**) megaspore mother cell (MMC) and aposporic initial (ai) at the same time; (**t**) megaspore tetrad after meiosis (MT) and aposporous embryo sac initial cell (ai); (**u**) 8-nucleate *Hieracium* type embryo sac with two polar nuclei (pn); arrows in (**q**,**u**) mark the micropyle. Black and white arrows mark the micropyle.

Flowers of *R. kuepferi* are initiated in the year preceding anthesis. Both at the natural sites and in the plots along the elevation gradient, floral apices at the earliest stage 1 were recognizable from July on in both cytotypes (Figure 1 and Figure 2b). Early floral preformation (stages 2–4, Figure 2c–e) proceeded independently of the current year flowering and fruiting (grey bars in Figure 1). At lower elevations where anthesis started early, floral development began about 3 weeks after fruit maturity. At higher elevations and in the natural stands, the first floral apices appeared during anthesis or ongoing fruit development. By the end of October, sepal, petal, stamen, and carpel primordia had partly or completely formed (stage 4–5, Figure 2f–h). Ovule primordia were not yet discernible. Flower buds of both cytotypes entered winter in a primordial, premeiotic stage at all sites. When emerging from the winter snow at the beginning of the growing season, terminal flower buds of 2*x* plants had considerable increased in size (5 times in length and 4.5 times in width on average; Figure 2i) and were usually in a premeiotic, meiotic (stage 6), or postmeiotic, prefloral stage (stage 7) irrespective of the date of thawing (see asterisks in Figure 1 indicating winter snow period). Stamina comprised short filaments, and, depending on site and cytotype, anthers contained all stages from the microspore mother cells to pollen tetrads and 1–2 celled pollen grains (Figure 2j–m). In parallel, carpels had elongated, and ovules were developing. At the natural sites and in the container plots up to P2600, ovules of thawing 2*x* plants were in the state of megasporogenesis (Figure 2n,o) or an early stage of megagametogenesis (Figure 2p). The mature embryo sac was of the eight-nucleate *Polygonum* type with two polar nuclei fusing into one (Figure 2q). As with 2*x* plants, terminal flower buds in thawing 4*x* plants had also considerably increased since autumn (Figure 2r). Most ovules (70%) showed traces of pre- to postmeiotic events and, at the same time, a laterally positioned aposporous initial cell (Figure 2s,t), which in about 15% of ovules had started to develop an unreduced embryo sac of the *Hieracium* type with two polar nuclei (Figure 2u). In P2800, ovules of both cytotypes were still in a primordial stage before the megaspore mother cell and, in 4*x* plants, an aposporous embryo sac initial cell had formed.

**Figure 3:**
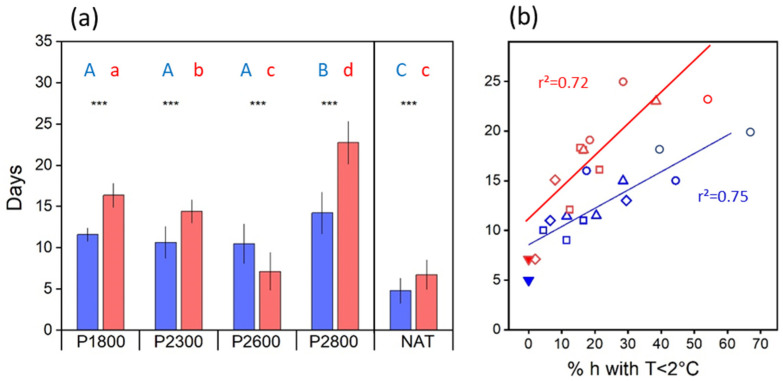
Duration of the prefloration period of single flowers of diploid (red) and tetraploid (blue) *R. kuepferi* plants at (**a**) different sites and (**b**) in relation to the frequency of hours at temperatures below +2 °C during the prefloration period. Bars in (**a**) represent means ± SD of all years of observation in the plots along the elevation gradient and at the natural sites (NAT). Different letters indicate statistical differences among sites within diploid (upper case letters) and tetraploid (lower case letters) plants (one-way ANOVA, Games–Howell post-hoc comparison). Asterisks indicate statistical differences between the cytotypes at the same site (*p* < 0.001, *t*-test). Symbols in (**b**) indicate plot means in different years for diploids (blue symbols) and tetraploids (red symbols) in the transect (open symbols) and at the natural sites (closed triangles). Open symbols: P1800 (upright triangle); P2300 (square); P2600 (diamond); P2800 (circle); closed triangle: NAT.

During the prefloration period (i.e., the period between spring snowmelt and the onset of anthesis), flower stalks elongated, and flower development was largely completed. The length of the prefloration period significantly differed (*p* < 0.001, *t*-test) between cytotypes at all sites (Figure 3a). At the natural sites, flowers of diploids opened 4–8 days (mean 4.8 ± 1.5 d), and flowers of tetraploids 5–10 days (mean 6.7 ± 1.9 d) after snowmelt (Figure 3a, NAT). In the transect, the prefloration period was up to four times as long as in plants at the natural sites. There was no uniform elevation trend. Between P1800 and P2600, the prefloration period was statistically the same in diploids but became significantly shorter (*p* < 0.001, one-way ANOVA, Games–Howell post-hoc comparison) in tetraploids. At the highest site, P2800, flower buds of both cytotypes took significantly (*p* < 0.001) longer to enter anthesis. These inconsistencies can be mainly explained by differences in temperatures during the prefloration period, which strongly depended on the time of snowmelt (see Figure 1). Earlier thawing plots at lower elevations experienced generally lower temperatures than later thawing plots higher up (Table S4). There was a clear relationship (r^2^ ≥ 0.72, *p* < 0.001) between the length of the prefloration period and the frequency of hours with a temperature below +2 °C (Figure 3b).

### 3.3. Duration of Anthesis and Seed Development on a Single Flower Basis

#### 3.3.1. Natural Sites

**Figure 4:**
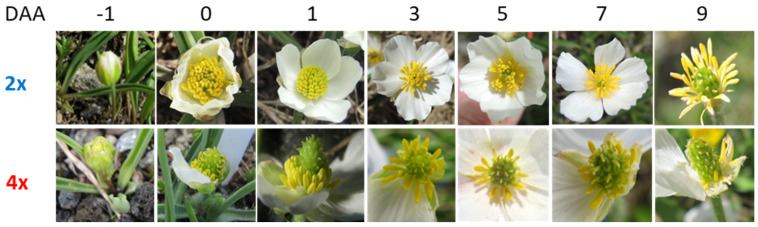
Sequence of anthesis in diploid (2*x*) and tetraploid (4*x*) *R. kuepferi* plants at the natural sites. Numbers indicate days after onset of anthesis (DAA). Sexual flowers of 2*x* plants are pentacyclic and form 32–85 stamens and 3–30 free carpels. In apomictic 4*x* flowers, sepal and petal numbers are irregular (0–5); the number of stamens (11–37) is much lower than the number of carpels (up to 100), which are arranged on a conical floral axis. The apocarpous gynoecium often bursts out of the still closed flower bud (see photo 4*x*, −1 d) and considerably overtops the stamens during anthesis. More details in the text.

**Figure 5:**
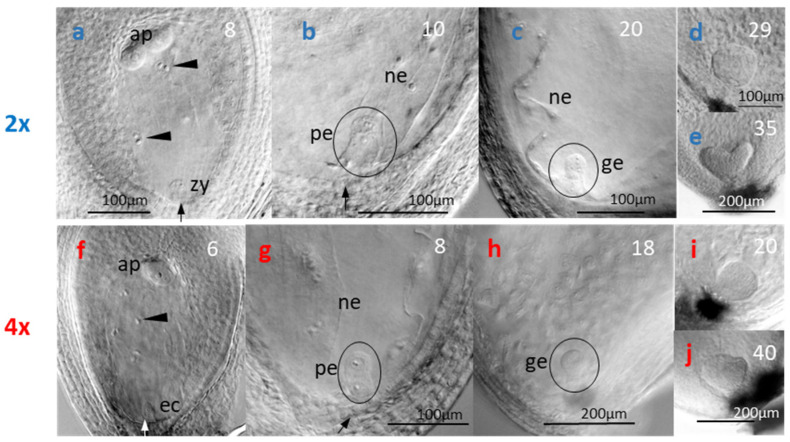
Seed development in (**a**–**e**) diploid sexual (2*x*) and (**f**–**j**) tetraploid apomictic (4*x*) *R. kuepferi* plants. White numbers on the right side indicate days after onset of anthesis (DAA). (**a**,**f**) first free endosperm nuclei (arrowheads), zygote (zy), and egg cell (ec), respectively; (**b**,**g**) nuclear endosperm (ne) and 2-celled proembryo (pe); (**c**,**h**) early globular embryo (ge); (**d**,**i**) late globular embryo; (**e**,**j**) heart-shaped embryo. Black and white arrows mark the micropyle.

**Figure 6:**
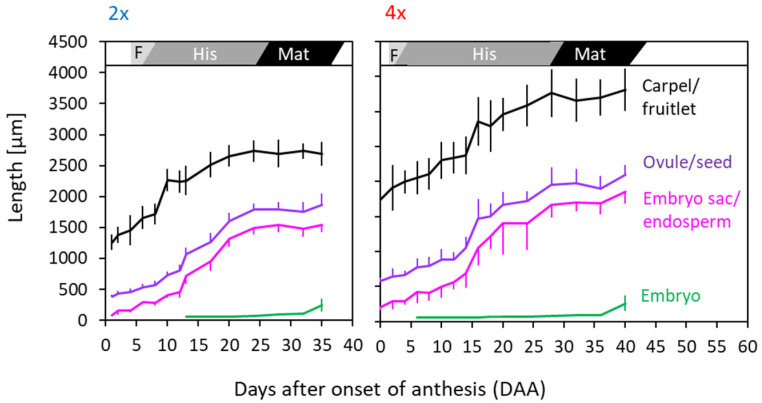
Dynamics of seed development in diploid (2*x*) and tetraploid (4*x*) *R. kuepferi* plants at the natural sites. F: Fertilization; His: Histogenesis; Mat: Maturation. Values are means; error bars indicate SD.

Diploid flowers are protandrous (Figure 4, 2*x*). Upon flower bud opening, anthers dehisced introrsely and dismissed pollen over a period of about 6 days after onset of anthesis (DAA). Petals wilted and were shed from day 9 on. During the male phase, stigmas became papillate, and within the ovules, megagametogenesis was completed. Pollinated stigmas and fertilized embryo sacs were observed from day 4 on. The 3n endosperm cell divided 6–8 DAA (Figure 5a). Zygote division started 8–10 DAA (Figure 5b). Histogenesis lasted for about 18 days, during which a nuclear (Figure 5b,c) and, later on, cellular endosperm (Figure 5d) were formed, and seeds greatly increased in size (Figure 6, 2*x*). At the end of histogenesis, embryos had reached an early globular stage and the cellularization of the endosperm had been more or less completed (Figure 5d). Seed maturation (reserve deposition and maturation drying) lasted for about 12 days. Mature seeds contained a late-globular or heart-shaped embryo (Figure 5e) and were surrounded by a light brown seed coat.

Tetraploid flowers were more or less protogynous. Papillous stigmas often emerged between still closed petals and were receptive from the beginning of anthesis (Figure 4, 4*x*); likewise, most ovules were ready for fertilization on day zero of anthesis and 2 DAA at the latest. Anthers released pollen grains between one and five DAA. On the second day of anthesis, most stigmas were covered with pollen grains, and in single embryo sacs, the first division of the fertilized central cell had taken place, initiating the formation of nuclear endosperm (Figure 5f,g). The unreduced, unfertilized egg cell divided after 4–6 DAA, resulting in a two-celled proembryo (Figure 5g). At that time, petals started to wilt, and flowers passed into the fruiting phase (Figure 4, 4*x*). During late histogenesis, the endosperm became cellular, and the embryo became globular (Figure 5h,i). Seed growth was completed after about 28 DAA, whereby fruitlets and seeds of the polyploid tetraploids grew markedly larger than those of diploid parent plants (Figure 6, 4*x*). Seed maturation took another 17 days, on average. At seed dispersal, apomictic embryos had reached a late-globular or heart-shaped stage (Figure 5j), and the seed coat was still greenish.

**Figure 7:**
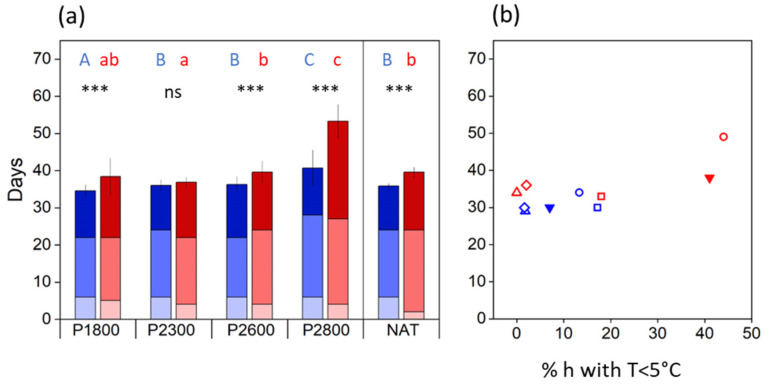
Postfloration period in *R. kuepferi* plants. (**a**) The full column length shows the mean duration ± SD of the total postfloration period in diploids (blue) and tetraploids (red) in the plots along the elevation gradient and at the natural sites (NAT). Sections within the columns stand for the period between onset of anthesis and fertilization (light colors), histogenesis (mid-tone), and maturation phase (dark colors). Different letters show statistically significant differences among the mean lengths of the total postfloration period at different elevations for diploid plants (upper case letters) and tetraploid plants (lower case letters) (one-way ANOVA, Games–Howell post-hoc comparison). Asterisks indicate significant differences between cytotypes at the same site (*** *p* ≤ 0.001, ns not significant, *t*-test). (**b**) Relationship between percent hours with temperatures below +5 °C and the mean duration of the total postfloration period at the different sites. Open symbols: P1800 (triangle), P2300 (square), P2600 (diamond), P2800 (circle); closed triangle: natural sites NAT.

Though seed development of diploids started later during anthesis, it proceeded considerably faster than in tetraploids (Figure 6 and Figure 7): from the beginning of anthesis until seed maturity, 2*x* flowers needed on average 35 days, and 4*x* flowers needed 40 days, which could be related to the differences in seed size. Counted from fertilization, the difference was even bigger, namely, 30 days in diploids and 38 d in tetraploids.

#### 3.3.2. Transect

In the transect, the sequence of flowering and seed development was about the same as reported for the natural sites. In single flowers, pollination and fertilization occurred within about 6 DAA in diploid flowers and within 4 DAA in tetraploid flowers, independently of elevation (Figure 7a). Histogenesis lasted between 16 d (2*x*) and 17 d (4*x*) at the lowest site and 22 d (2*x*) and 23 (4*x*) at the highest site. Within a cytotype, seed maturation took about the same time at all elevations in 2*x* plants (12–14 d) and up to P2600 in 4*x* plants (16–17 d). At P2800, however, tetraploids took considerably longer (26 d) to mature seeds. Thus, depending on elevation, the whole postfloration period from onset of anthesis until seed maturation took, on average, 34–40 d in 2*x* flowers and 36–53 d in 4*x* flowers. Except for P2300, the differences between cytotypes were significant (*p* < 0.001, *t*-test) at each site. Site temperatures and the frequency of hours below +5 °C during the whole period can only partially explain the difference in the postfloral period lengths between 2*x* and 4*x* plants (Figure 7b). Intermittent short cool periods had no noticeable effect on the development time. In over 40% of hours, however, low temperatures increasingly mattered, as observed in 4*x* plants at P2800. Because of a longer prefloration period (see Figure 3a), flowering started later in the season, and seed maturation shifted into September when night temperatures regularly fell below +5 °C.

### 3.4. Male Performance

#### 3.4.1. Anthers

**Figure 8:**
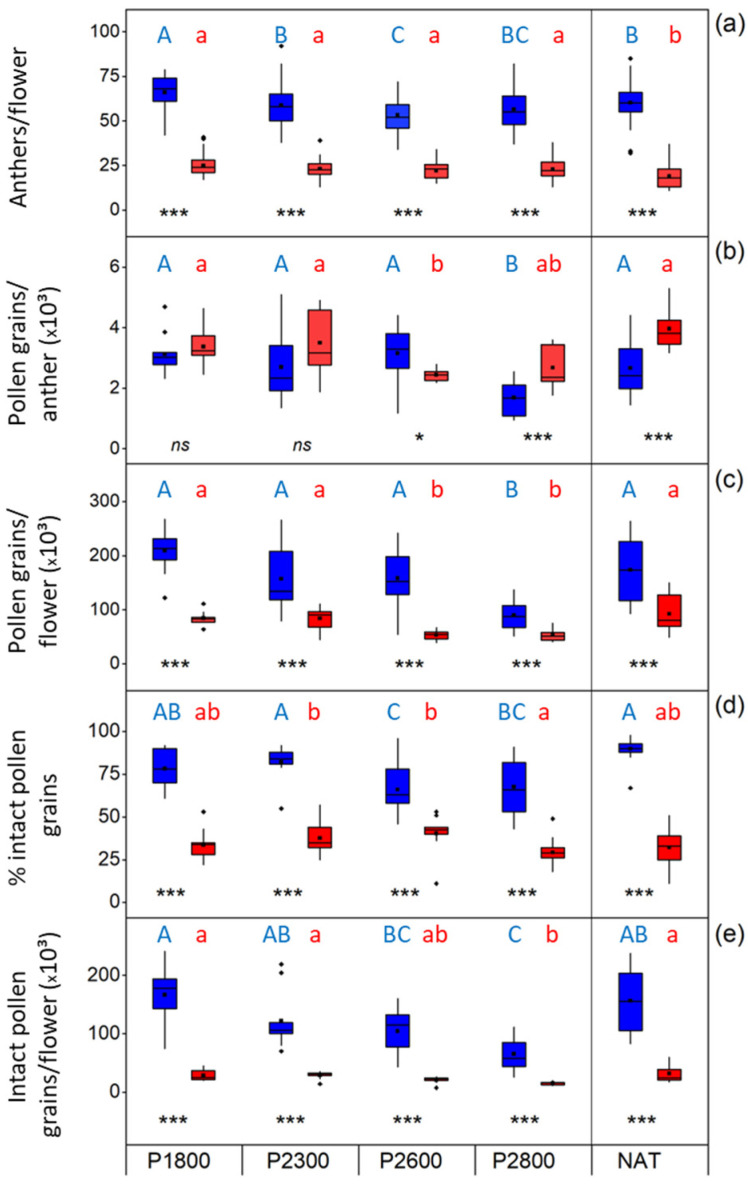
Male performance of diploid (blue) and tetraploid (red) *R. kuepferi* plants along the transect and at the natural sites (NAT) in terminal flowers. (**a**) number of anthers per flower; (**b**) total number (intact and defect) of pollen grains per anther; (**c**) total number of pollen grains per flower; (**d**) pollen viability expressed as percentage of intact appearing pollen grains; (**e**) number of intact pollen grains per flower. Boxes show the median (line inside the box), the mean (square symbol inside the box), the 25th and 75th percentile (extent of box), maximum and minimum values within the 1.5 times the interquartile range (whiskers), and outliers (diamonds). Different letters below the boxes show statistically significant differences among different elevations for diploid plants (upper case letters) and tetraploid plants (lower case letters) (one-way ANOVA, Duncan post-hoc comparison). Asterisks indicate significant differences between cytotypes at the same site (* *p* ≤ 0.05, *** *p* ≤ 0.001, ns not significant, *t*-test).

Diploids developed more than twice as many stamens per terminal flower than tetraploids at all sites (Figure 8a). Along the transect, anther number slightly decreased with elevation in 2*x* flowers but not 4*x* flowers. In the natural populations, 2*x* plants developed about the same number of anthers as individuals in the plots P2300 and P2800, significantly fewer (*p* = 0.004, *t*-test) than individuals in P1800 but significantly more than in P2600 (*p* < 0.001); naturally growing 4*x* plants, however, formed fewer anthers than individuals along the whole transect (on average 19 vs. 22–25).

#### 3.4.2. Pollen

Along the transect, pollen grain number per anther was within the same range in both cytotypes at the lower sites but decreased above P2600 and P2300 (Figure 8b). The decrease was more pronounced in diploids than in tetraploids. Nevertheless, because of the higher anther number (Figure 8a), diploids developed significantly more pollen grains per flower than tetraploids at all sites (*p* < 0.001, *t*-test) (Figure 8c). Up to P2600 (2*x*) and P2300 (4*x*), pollen grain number per anther and flower did not differ significantly from that of the individuals at the natural sites but was significantly lower at higher sites.

Pollen viability was considerably higher in diploids than in tetraploids both along the transect and at the natural sites (Figure 8d; Figure S7 for microscopic imaging). Mean pollen viability amounted to 90% (natural population) and 66–82% (transect) in 2*x* plants, 32% (natural population), and 29–40% (transect) in 4*x* plants. In tetraploids, most defective pollen grains had stopped development in the microspore stage or during male gametogenesis. Pollen viability slightly decreased with elevation in 2*x* plants but not 4*x* plants.

Because of the higher number of anthers (Figure 8a) and the higher pollen viability (Figure 8d), 2*x* plants produced, depending on the site, four to six times more intact pollen grains per flower than 4*x* plants (Figure 8e). The number of intact pollen grains substantially decreased with elevation in diploids but only marginally in tetraploids. At the natural sites, pollen viability showed a similar distribution range as in the lowest transect plots.

**Figure 9:**
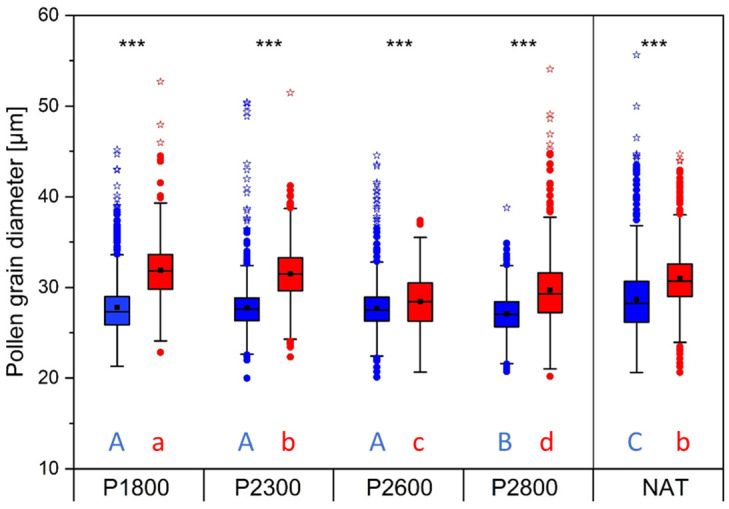
Pollen grain diameter of diploid (blue) and tetraploid (red) *R. kuepferi* plants at different elevations and the natural sites (NAT). For details on the boxes, see Figure 8. In Figure 9, closed circles indicate outliers exceeding the 1.5-fold box length, and asterisks indicate extreme values exceeding the 3-fold box length. Depending on site and cytotype, on average, 1280 pollen grains in 25 diploid flowers and 850 Pollen grains in 18 tetraploid flowers were measured. Different letters below the boxes show statistically significant differences among the means at different elevations for diploid plants (upper case letters) and tetraploid plants (lower case letters) (one-way ANOVA, Games–Howell post-hoc comparison). Asterisks indicate significant mean differences between cytotypes at the same site (*** *p* < 0.001, *t*-test).

Viable appearing pollen grains of both cytotypes varied considerably in size (Figure 9). Pollen grains were significantly smaller in diploids than in tetraploids at all study sites (*p* < 0.001, *t*-test), which also became apparent in the different size class modes (2*x*: >24–28 µm, 4*x*: >28–32 µm) (Figure S8, dark colors). Along the transect, pollen grains in diploids hardly changed in size, whereas those in tetraploids were significantly larger at lower elevations than at higher elevations (one-way-ANOVA, Duncan post-hoc comparison, *p* < 0.001). In both cytotypes, giant, vital appearing pollen grains up to 55 µm occurred (Figure S7a–c; extreme values in Figure 9 and Figure S8). In relation to the total number of pollen grains measured per site, the proportion of giant pollen grains was mostly below 1% (Figure S8, dashed lines), except in the 2*x* NAT plants. The proportion of 2*x* flowers containing larger grains decreased along the transect, was zero at P2800, but was remarkably high in 2*x* NAT plants (Figure S8, light blue area). In 4*x* plants, only a few flowers contained larger pollen grains at the lower transect sites, but conspicuously many at the highest site P2800 (Figure S8, light red area).

### 3.5. Female Performance

**Figure 10:**
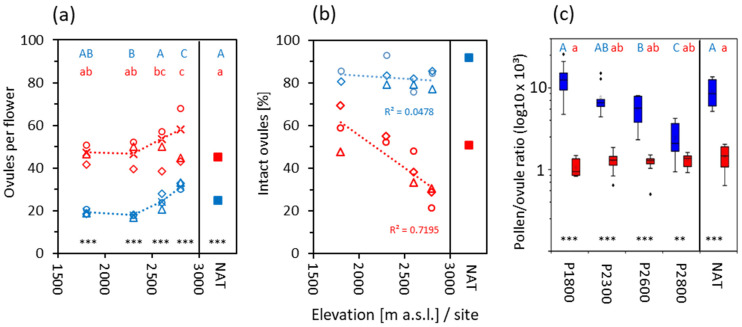
(**a**) Number of ovules per terminal flower and (**b**) percent intact ovules in year 1 (triangle), year 2 (diamond), and year 3 (circle) following transplantation in the elevation transect and at the natural sites (NAT) in diploid (blue) and tetraploid (red) *R. kuepferi* plants. Open symbols show yearly means; cross symbols with the dotted line in (**a**) show three-year means of all single values at each elevation. (**c**) Pollen/ovule (P/O) ratio calculated from the number of intact pollen grains and intact ovules of terminal flowers. For details on the boxplots, see Figure 8. Different letters indicate significant differences in three-year means among elevations within diploids (blue upper-case letters) and tetraploids (red lower-case letters) (one-way ANOVA, Games–Howell post-hoc comparison). Asterisks indicate significant differences between the cytotypes at the same elevation or between natural populations (** *p* ≤ 0.01, *** *p* ≤ 0.001; *t*-test).

Tetraploids developed about twice as many carpels per terminal flower than diploids both at the natural sites and in the transect plots. Since carpels of *R. kuepferi* are uniovular, these values also apply to the number of ovules per flower (Figure 10a). At the same transect site, ovule number per flower hardly differed among 3 investigation years in 2*x* plants but greatly varied in 4*x* plants. Most striking was the increase in the ovule number with elevation in both cytotypes. In NAT diploids, ovule numbers corresponded to those found at P2600. In NAT tetraploids, values were in the range of P1800 and P2300 plants but remained significantly (*p* < 0.008, one-way ANOVA, Games–Howell post-hoc comparison) below the values at the highest site.

**Figure 11:**
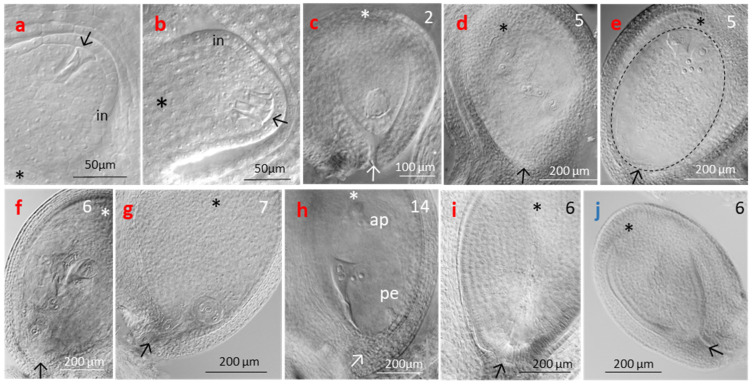
Developmental disturbances during ovule development in (**a**–**i**) tetraploid and (**j**) diploid *R. kuepferi* plants. (**a**,**b**) young ovule showing developmental disorder during an attempted megasporogenesis and aposporous embryo sac development in the micropylar region (arrow) of the nucellus; the integument (in) is still primordial. (**c**) embryo sac development had stopped in the 2-nucleate stage. (**d**) embryo sac nuclei in a chaotic arrangement. (**e**) huge embryo sac (broken line) missing the egg apparatus and functional nuclei in the central cell. (**f**,**g**) collapsed embryo sacs. (**h**) ovule 14 d after onset of anthesis with an apomictic proembryo (pe) but missing endosperm development. Embryo sack lacking ovules from (**i**) diploid and (**j**) tetraploid plants. Arrows mark the micropylar, and asterisks mark the chalazal region. Numbers at the upper right side indicate days after onset of anthesis (DAA).

In diploids, 75–90% of the ovules at the natural sites and in the transect contained well-developed embryo sacs (Figure 10b). In tetraploid NAT plants, on average, only 52% of ovules were intact; along the transect, the proportion of intact ovules linearly decreased with increasing elevation from 48–69% to 20–30% (r^2^ = 0.72, *p* = 0.001). Anomalous ovules of tetraploids showed developmental disorder around megasporogenesis and the formation of an aposporous initial (Figure 11a,b), arrested embryo sac development (Figure 11c), embryo sac nuclei in a chaotic arrangement (Figure 11d), huge embryo sac missing the egg apparatus and functional nuclei in the central cell (Figure 11e), collapsed embryo sac (Figure 11f,g), embryo sac with already developed proembryos but lacking endosperm development (Figure 11h), and embryo sac completely missing (Figure 11i). Dysfunctional ovules of diploids usually lacked embryo sacs (Figure 11j), obviously due to problems during meiosis or early megagametogenesis.

### 3.6. Pollen/Ovule Ratio

At the natural sites, the mean ratio of the total number (intact plus malformed) of pollen grains (P) to ovules (O) per terminal flower amounted to 9200 in diploids and 2100 in tetraploids. Considering only the fraction of intact appearing pollen grains (Figure 8d, NAT) and ovules (Figure 10b, NAT), the P/O ratio per flower was 9000 in diploids and 1440 in tetraploids (Figure 10c, NAT). Along the transect, the mean P/O ratio in diploids drastically decreased from 14,000 (total) and 13,400 (intact) at the lowest site to 2800 (total) and 2500 (intact) at the highest site (r^2^ = 0.55, *p* < 0.001 in both cases); the marked decline results from the decreasing number of viable pollen grains with elevation (Figure 8e) in relation to the increase in ovules per flower (Figure 10a). In tetraploids, the mean P/O ratio declined to a lesser degree from 2000 at the lowest site to 1200 at the highest site when calculated from the total number of pollen grains and ovules (r^2^ = 0.3, *p* < 0.001) but did not change significantly (r^2^ = 0.08, *p* = 0.07) along the transect when only intact structures were considered. In this case, the number of pollen grains per flower did not substantially change with elevation (see Figure 8e), whereas the increasing number of ovules (Figure 10a) partly compensated for the increasing proportion of defect ovules (Figure 10b). Thus, the P/O ratio remained largely the same throughout the transect.

### 3.7. Stigma Pollen Load, Pollen Germination, Pollen Tube Growth, and Reproductive Success

Stigma pollen load (i.e., the number of conspecific pollen grains per stigma) in naturally pollinated flowers was highly variable in both cytotypes at all sites (Table 1A). At lower elevations, significantly more pollen grains were deposited than at higher elevations (*p* < 0.001, one-way-ANOVA, Duncan post-hoc comparison). Though in 4*x* flowers, up to 70% of pollen grains were small and malformed, defect grains were hardly observed on stigmas. Obviously, they did not adhere to the stigma but dropped down or were washed away by rain. Thus, the stigma pollen load consisted almost exclusively of intact appearing pollen grains in both cytotypes. In diploids, most deposited pollen grains germinated (Table 1B), resulting in a high germination percentage (72–78%) at all elevations (Table 1C). But, if any, only a few pollen tubes grew down the style irrespective of the initial pollen load (Table 1D). The mean seed set (39–56%), due to a high variation among individual flowers, did not significantly change along the transect (Table 1E). The same holds true for the mean seed number of 10–11 per flower, except for the lower seed output in P2300 (Table 1F). In tetraploids, substantially fewer pollen grains germinated, and still fewer grew pollen tubes (one–two). The low number of pollen tubes per carpel, together with declining ovule integrity along the transect (see Figure 10b), matches well with the low seed set of only 7% and a mean output of four seeds per flower at the highest site (Table 1E,F). At the natural sites, seed set and seed number per flower were significantly higher than in the transect both in diploids (seed set: *p* ≤ 0.001, except for *p* = 0.276 when comparing NAT with P1800 plants; seeds: *p* ≤ 0.008) and in tetraploids (seed set and seeds: *p* ≤ 0.001) (one-way-ANOVA, Games–Howell post-hoc comparison). Despite a significantly lower seed set in tetraploids (*p* ≤ 0.001, *t*-test), the seed number was statistically the same in both cytotypes (*p* = 0.57, *t*-test), which can be explained by the average carpel number being twice as high in tetraploids than in diploids.

### 3.8. Reproductive Mode of Diploid and Tetraploid Plants at Different Elevations

Flow cytometric seed screening showed no clear influence of the site climate on the mode of reproduction. At all elevations, diploids formed predominantly sexual seeds (98–100%), whereas tetraploids had apomictic seeds (92–98%; Table S3). There was no significant correlation of either reproductive mode to elevation (*p* = 0.591 for diploids and *p* = 0.938 for tetraploids). Apomictic seeds were all formed via pseudogamy, whereby a predominant peak index of c. 2.5 indicated that mostly one sperm nucleus fertilized the polar nuclei (embryo 4Cx, endosperm 10Cx).

## 4. Discussion

As outlined in the introduction, ecological data and simulations of the Holocene range dynamics of the sexual–apomictic mountain plant *Ranunculus kuepferi* revealed, on the basis of environmental descriptors, primarily a niche shift of the apomicts toward colder conditions [55,56]. Climate chamber experiments and epigenetic data [57,64,65], as well as freezing tolerance tests on plants growing in an alpine elevation gradient [59], confirmed a better cold acclimation of tetraploids in vegetative parts. The observed niche shift led us to expect that in common garden experiments along the temperature gradient from the subalpine to the subnival zone, reproductive fitness would decrease more in sexual diploids than in tetraploid apomicts. However, our results did not reflect such a trend. On the contrary, diploids mostly performed better than tetraploids at all elevations, as well as in the natural populations.

### 4.1. Flower Preformation

In *R. kuepferi*, flower bud initiation occurs in the growing season preceding the year of anthesis. In both cytotypes, floral development started nearly synchronously in July at all elevations, independently of the time of anthesis and fruit development. This suggests a photoperiodic trigger of floral induction (decreasing long day conditions), as found in other mountain plant species [66,67,68]. Early development proceeded similarly in both cytotypes; however, primordia differed in their developmental fate in that tetraploid flowers differentiated about twice as much carpels and correspondingly fewer stamens than diploid flowers. Furthermore, the number of carpels significantly increased with elevation in both cytotypes, which indicates a strong impact of the environmental conditions at the time of the initiation of carpels and, thus, ovules. According to the concept of parental optimism, over-investment in the relatively inexpensive early stages of offspring, such as ovules, is favored, particularly in climatically unpredictable environments [69]. Ovule oversupply as a variant of bet-hedging strategy allows plants to take advantage of the rare occasions of abundant pollen receipt in stressful habitats [70] and has been evidenced for a number of plant species along an elevation gradient in different mountain systems on earth [71,72,73,74,75,76]. In *R. kuepferi*, more ovules at higher elevations may be of different significance for both cytotypes. For the outcrossing diploids, which are dependent on insect pollination, more ovules increase the possibility of a higher seed output in climatically favorable growing seasons. For self-fertile apomicts, however, a greater ovule number may partly compensate for the losses due to the increasing developmental disturbances with increasing elevation.

At the start of winter conditions in late autumn, preformed flower buds of both cytotypes were still small and underdeveloped. Ovule primordia had not developed yet, and anthers were in an archesporial state, which corresponds to the winter state in a number of high mountain plants investigated up to now [68,77]. At snowmelt in the mountain spring, starting between May and early July along the transect, buds had markedly increased in size and were in a pre- to post-meiotic state of pollen development and at the beginning of the embryo sac development. Developmental progress must have occurred under the winter snow cover at uniform soil surface temperatures around zero, according to the temperature records in all plots. We presume that continued flower bud development did not proceed steadily during the whole winter time but started in late winter when the package of crusted snow began to soften, and water penetrated the soil. Subnivean development is widespread among perennial plants inhabiting cold regions [78,79] and, thus, increases the length of their growth season [80]. Thawing at higher elevations in June and July proceeds more quickly than at lower elevations in May, when night frosts occur more frequently [81], which explains why flower buds emerging from the snow were more advanced at higher than at lower elevations.

Microscopic observation of embryo sac development in tetraploids showed a high proportion of ovules with traces of possible incomplete meiotic events and a larger, somewhat laterally positioned aposporous initial cell. Mixed ovules can either be the result of unsuccessful female meiosis or a facultative dominance of the apomictic pathway interrupting the sexual pathway [24,82]. During embryo sac development, an unreduced aposporous initial cell might have a further competitive advantage over the reduced megaspore due to the double chromosome complement [82]. In any case, the parallel formation of sexual and/or apomictic initials enables switching between both reproductive modes, which has been evidenced by flow cytometric screening of seeds collected in different tetraploid *R. kuepferi* populations [46,51].

As stated in the introduction, the dominance of the sexual or the apomictic way seems to be additionally influenced by environmental factors [16,29,30,31]. In a climate chamber experiment on *R. kuepferi* with repeatedly moderate nocturnal frost (−1 °C), frost-treated diploid plants produced significantly more apomictic seeds than non-frost-treated plants, whereas the tetraploids showed no difference between treatments [16]. In the present transect study, we observed for diploids one apomictic seed each at P1800 and P2600 (c. 1–2%), which resembles the spontaneous low frequencies of apomixis in natural diploid populations in the Southwestern Alps [46]. However, there is no apparent effect of elevation on the mode of reproduction. In tetraploids, our data also fit into the range of rare sexuality at elevations between 2400 and 2700 m [46] and of insignificant differences in cold/warm treatments [16].

A major problem of tetraploids is the high proportion of ovules with developmental disorders (embryo sac lacking or dysfunctional), indicating instability in the genetic, epigenetic, and transcriptional regulation [18,19,82]. In autopolyploid plants, this instability probably affects sexual ovules more than apomictic ones due to more irregular meiosis, potential multivalent formation, and unequal segregation of chromosomes, e.g., [83]. These irregularities, together with the haploid condition of embryo sacs, cause higher abortion rates of the sexual ovules, finally resulting in relatively higher proportions of the few surviving apomictic ovules and seeds [82]. Disorders in tetraploids increased with elevation from about 40% in the subalpine plots up to 70% in the subnival plots. Conversely, in diploids, the proportion of ovules lacking embryo sacs remained nearly constant at 20% over the whole elevation gradient. This would mean that the apomictic pathway is more prone to interference from environmental conditions than the meiotic pathway. Since embryo sac development starts around snowmelt in spring and proceeds during the prefloration period, elevation-specific conditions at that time might influence its development in tetraploids. Most plausible appear daily minimum temperatures during the prefloration period, which were 2–8 °C at the subalpine and the alpine sites, 1–5 °C at the upper alpine site, and around zero and below at the subnival site. In tetraploids, such low-temperature regimes at higher elevations might have negative effects on the strongly precocious aposporous embryo sac development by disturbing mitoses (see Figure 11). That is, the advantage of precocious embryo sac development postulated by [5] would become a disadvantage at frost-dominated sites.

Not only ovule development but also pollen development was considerably disturbed in tetraploids, as already shown in previous studies [11,60]. Percent viability varied according to the sample origin and viability test used, but in all studies, including ours, it was significantly lower in tetraploids than in diploids. However, as for embryo sac development, we found an additional site effect suggesting an influence of temperature conditions during meiosis and pollen development, which occurs around the time of snowmelt within a few days. Cold temperatures may provoke tapetal irregularities, cytoskeletal alterations, and various metabolic problems, which disturb microsporogenesis and male gametogenesis [84,85]. This is exactly what we observed: defective pollen grains had stopped development either in the microspore stage or later during microgametogenesis.

Adverse environmental conditions such as cold stress do not always have to result in a breakdown in pollen development. Alterations in the formation and organization of the meiotic cytoskeleton can also lead to incorrect chromosome segregation and aneuploidy [85] or to genome multiplication, which results in the production of unreduced, diploid, or polyploid gametes ([84,86,87] for reviews). In most cases, polyploid pollen grains arise through meiotic restitution but can also result from pre-meiotic and post-meiotic genome doubling [15]. Generally, a bimodal frequency distribution of pollen size could be a possible indication for unreduced pollen grains. Irregularly sized and abnormally large pollen grains containing larger or multiple nuclei may indicate a previous genome amplification. In the case of *R. kuepferi*, pollen size strongly varied in both cytotypes, as already reported earlier [11]. We found intact appearing pollen grains in diploids peaking at a lower size class (>24–28 µm) than in tetraploids (>28–32 µm). In some flowers, a small proportion of normally structured grains exceeded the peak size by up to two times. We did not find a discernible effect of site temperatures on the frequency of giant pollen grains. However, giant grains were found relatively frequently in flowers of the diploid natural population, which might indicate a high intrinsic potential of restitutional meiosis in the sexual parental population. Our FCSS data, however, do not indicate that unreduced pollen was involved in seed formation. For *R. kuepferi*, several pollen size classes greater than the modal value rather speak for mainly unbalanced meiosis, leading to different states of aneuploidy and disturbances during male gametophyte formation [24].

### 4.2. Anthesis

In general, anthesis starts with the opening of the corolla and male (protandrous) or female (proterogynous) organs, or both at the same time (homogamous) become functional. In *R. kuepferi*, the timing of sexual functions clearly reflects the cytotype-specific mating system. The outcrossing diploids started with the male phase during which stigmas became successively papillated, and megagametogenesis was completed within the ovules. Self-fertile tetraploid flowers, however, are homogamous or slightly protogynous. The sequence of development seemed to be disturbed in that papillous stigmas emerged between closed petals in flower buds near the ground before bolting. Stigmas were papillate and receptive from the beginning of anthesis, and most ovules were ready for fertilization of the secondary nucleus in the apomictic embryo sac. In line with the cytotype-specific mating system is the pollen/ovule ratio. The ratio is high in diploids, which is typical for outcrossers, and significantly lower in apomicts, which is typical for selfers [88]. As outlined above, the basis for this ratio is laid during the early flower ontogenesis when the number of stamens and carpels is fixed. Interestingly, the number of intact pollen grains per flower declined substantially with elevation in diploids, partly because of a reduction in anthers and partly because of a decrease in intact pollen grains. In apomicts, by contrast, there was no elevation effect regarding the number of stamens and hardly any change in the principal high number of dysfunctional pollen grains per flower. Nonetheless, even at the highest site, there was still an excess of pollen grains by 1300-fold in diploids and 1000-fold in apomicts in relation to the number of ovules. Thus, pollen limitation could be theoretically excluded in selfing tetraploids, whereas the pollination success in diploids depends on the efficiency of the pollen transfer by insects. Data for the stigma pollen load, however, showed a more differentiated picture. In both cytotypes, stigma pollen load was in a similar range, with a maximum at lower sites and a significant decrease toward higher sites (see Table 1A). This result suggests that *R. kuepferi* was partly pollen-limited at the higher sites and, furthermore, that autodeposition must have played a minor role in tetraploids. Indeed, apomictic flowers appeared to some extent herkogamous in that anthers were in a lower position than most stigmas (see Figure 4). This would mean that flowers of both cytotypes depend on insect visitation - in diploids for cross-pollination and, in tetraploids, for selfing. The frequency of visitors was rather low in the natural populations of both cytotypes (U. Ladinig, pers. obs.). Flower visitors were mainly flies but also syrphides and ants, whose carry-over efficiency is rather low [89,90]. In occasional observations along the transect, the visiting rate appeared even lower than at the natural sites and decreased with elevation, which would explain the decreasing pollen load in flowers of both cytotypes.

Pollen grains germinated at more than 70% in diploids but only around 40% and less in tetraploids, which was in line with pollen viability, microscopically assessed by the acetocarmine test. Remarkably, even in carpels with a relatively high number of germinated pollen grains, only a few of them formed pollen tubes, which, in apomicts, often stopped growing in the style. Possible reasons are competition for space and/or maternal resources [91] but also a lack of proper chemical guidance signals in the transmitting tract, the ovary, and the ovule [92]. It is thinkable that weak or improper signals could be a problem in the male–female crosstalk in apomicts where several floral functions are inherently impaired.

### 4.3. Seed Development

As outlined before, functional apomictic embryo sacs were usually mature at the beginning of anthesis, whereas meiotic embryo sacs of diploids matured only during the male phase. Due to this delay, we expected a longer post-floral development in diploids than in tetraploids. In reality, seed development proceeded significantly quicker in diploids than in tetraploids, both at the natural sites and along the elevation transect. The developmental delay in apomictic seeds also became apparent in the incomplete maturation of the pericarp. Apomictic achenes not only remained longer attached to the receptacle but were mostly released in a green state. In contrast, sexual achenes of the diploids were shed as soon as the pericarp had turned brown. Again, these observations suggest imbalances in the development program of seeds, which might, inter alia, be caused by an imbalance between the male and female genome in the endosperm [93,94]. Our FCSS data revealed predominantly 10C*x* endosperms, indicating a surplus of eight maternal genome contributions to two paternal genome contributions (=4:1).

The high proportion of defective ovules and further abortions during seed development resulted in a rather low seed output in apomicts, particularly at higher sites. Even the higher number of carpels per flower could not compensate for the losses in this case. In the natural 2*x* and 4*x* populations, however, the mean seed output per flower did not differ statistically (see Table 1F). Here, the number of fertile ovules was roughly the same in both cytotypes because twice as many ovules in 4*x* flowers compared to 2*x* flowers compensated for the about 50% failure rate. Thus, the absolute reproductive success could be the same in apomictic and sexual natural populations. If so, the number of offspring is not necessarily a key criterion for the dispersal potential of the cytotypes. Rather, a higher seed mass [56,59] and a higher potential for forming seed banks [59] could be important advantages for the establishment of tetraploid populations.

### 4.4. Developmental Dynamics

Temperatures generally influence the length of generative development as reported for a number of arctic and alpine plant species, e.g., [95,96,97,98]; however, the effect depends on the development phase and the temperature range [68]. In *R. kuepferi*, low-temperature effects were particularly pronounced in the prefloration period (see Figure 3b). This developmental phase was specifically short at late melting sites where, during subsequent summer conditions, night temperatures seldom dropped below 5 °C but took nearly four times longer at earlier melting sites where the percentage of hours ≤2 °C was high. Interestingly, the approximately 1:1.5 duration ratio between diploids and tetraploids remained largely unchanged throughout the entire time–temperature range. This points to a genetically based retardation in the final development of apomictic flowers, including the process of bolting compared to diploids. Seed development was less affected by low temperatures because this developmental phase mainly occurred during summer from June to mid-August, except for the highest site, P2800. Here, seed maturation was delayed until September because of generally lower temperatures at that time, particularly during the night.

Site climate exerts a significant impact on the total length of the reproductive period. According to the date of snowmelt and, subsequently, the elevation-dependent temperature conditions, the lengths of the developmental sub-phases within an individual flower may vary within a species-specific time frame, which can range from 40 d to more than 90 d in different mountain species [68]. The reproduction time of *R. kuepferi* ranks in the lower to middle range, whereby diploids required less time to produce mature seeds (41–56 d) than tetraploids (47–62(79) d). Remarkably, the shortest period within each cytotype was found in natural populations, suggesting climatic optimum conditions at the respective sites. This applies particularly to the diploid populations in the Western Alps. Because of moist Mediterranean air masses, characterized by mild temperatures and plentiful snow in winter, snowmelt is rather late, and during the warm summers, night temperatures seldom drop below 5 °C. The site climate at plot site P2600 was similar, where late snowmelt and balanced summer temperatures offered better conditions for growth and development than at lower sites where, due to the early snowmelt, temperatures at the beginning of the growing season were still moderate.

## 5. Conclusions

Contrary to our expectations, diploids showed a better overall performance than apomicts in the common garden study along a climatic elevation gradient. Developmental disturbances during floral ontogenesis caused reduced male and female gametophyte fertility, and abortions during seed development increased with elevation and led to a considerably lower seed output in tetraploids than in diploids at the highest sites. Furthermore, the longer pre- and postfloration periods in tetraploids jeopardize seed maturation, particularly at late thawing sites higher up; while seeds of diploids matured in time in late August, seed maturation in tetraploids was prolonged far into September when day length rapidly decreased and the frequency of snow days and night frosts steadily increased. This means that tetraploids, rather than diploids, in addition to the losses by seed abortion, are at risk of losing parts or the whole seed crop when the growing season ends too early. On the other hand, late seed deposition could avoid heat exposure and drying of seeds when laying on the soil surface in the hot summer months (July/August); in general, seeds stored under cool conditions have better germination rates in *Ranunculus* (E. Hörandl, pers. obs.).

However, tetraploids showed single developmental features which could be beneficial under certain conditions. Since peduncle elongation is one of the most vulnerable processes in the reproductive cycle of a plant [81], delayed bolting and corolla opening might prevent flowers from frost damage at the time of winter snowmelt when—due to surrounding snow fields—night temperatures may still drop below zero. Self-pollination seems not to be very effective without the aid of flower visitors in apomicts. Nevertheless, even a few self-pollinated carpels have the chance to produce at least a few seeds in the absence of pollinators, which would not be the case in the self-sterile sexuals, as observed in [42]. In this context, Baker’s law [45] becomes effective, saying that in the case of uniparental reproduction, even one seed can theoretically found a new population in contrast to outcrossing sexuals, which need both mating partners and pollen vectors. This advantage not only allowed for a faster recolonization of previously glaciated areas [56] but was also more efficient for long-distance dispersal, explaining the occurrences of tetraploids only on Corsica and in the Apennines [51]. Similar GP patterns are observed in the *Ranunculus auricomus* complex, in which tetraploid apomicts obtained much larger distribution areas than diploids despite lower seed sets and lower pollen quality [47]. In *R. kuepferi*, the better cold acclimation of tetraploids in vegetative parts [57,59,64,65] probably enabled the polyploids to survive the niche shift to colder climates in the Alps despite lower reproductive performance.

To conclude, the combination of various specific features reported here and in a parallel report [59] might have compensated for the partly defective reproductive development of apomictic tetraploids and, thus, might have promoted the vast expansion across the Alps.

## Figures and Tables

**Table 1:** Mean number ± SD of (**A**) pollen grains on a single stigma, (**B**) germinated pollen grains, (**C**) % germinated pollen grains, (**D**) number of pollen tubes per style, (**E**) seed set, and (**F**) number of intact seeds per flower in naturally pollinated diploid (2*x*) and tetraploid (4*x*) *R. kuepferi* plants along the elevation gradient.

		(A)	(B)	(C)	(D)	(E)	(F)
Cytotype	Site	Stigma Pollen Load	Germinated Pollen Grains	% Pollen Grains Germinated	Pollen Tubes per Style	Seed Set %	Seeds per Flower
2*x*	P1800	41.8 ± 35.2 a	34.6 ± 30.5 a	75 a	1.8 ± 1.0	56 ± 27 ab ***	10.7 ± 6.8 ab ns
	P2300	54.7 ± 34.9 a	44.4 ± 31.1 a	78 a	3.2 ± 1.5	40 ± 23 a **	6.7 ± 4.3 b ***
	P2600	8.1 ± 10.2 b	6.6 ± 7.8 b	72 a	3.0 ± 2.0	42 ± 22 a ***	9.8 ± 6.5 a ***
	P2800	12.4 ± 13.8 c	10.2 ± 12.7 b	72 a	1.5 ± 0.7	39 ± 25 a ***	11.3 ± 8.5 a ***
	NAT					68 ± 20 b ***	16.5 ± 7.0 c ns
4*x*	P1800	13.7 ± 11.2 a	7.3 ± 8.1 a	48 a	2.0 ± 0.0	19 ± 14 a	8.8 ± 6.7 a
	P2300	53.7 ± 28.8 b	23.2 ± 13.9 b	43 a	2.1 ± 0.9	27 ± 14 b	11.5 ± 6.0 a
	P2600	5.6 ± 5.4 c	1.5 ± 2.1 c	25 b	1.0 ± 0.0	10 ± 9 c	5.5 ± 6.1 b
	P2800	2.5 ± 4.1 c	1.7 ± 1.9 c	42 a	1.0 ± 0.0	7 ± 6 c	4.1 ± 3.8 b
	NAT					40 ± 17 d	18 ± 10 c

Transect data in A–C refer to single carpels (*n* = 39–102 per site and cytotype) from terminal flowers (n = 8–15 per site and cytotype). The number of pollen tubes (D) was ascertained in up to 15 carpels per site. Seed output (E, F), depending on availability, was determined in 29–109 (2*x*) and 42–71 (4*x*) aggregate fruits in the transect and 70 (2*x*) and 95 (4*x*) aggregate fruits in the natural populations. Different letters in each column within a cytotype indicate significant differences among different sites along the elevation gradient (one-way ANOVA, Duncan post-hoc comparison). Asterisks below diploid values indicate significant differences against tetraploids at the same elevation or between natural populations (** *p* ≤ 0.01, *** *p* ≤ 0.001; ns not significant, *t*-test).

## Data Availability

The data presented in this study are available upon request from the corresponding author.

## Supplementary Materials

The following supporting information can be downloaded at https://www.mdpi.com/article/10.3390/life14091202/s1, Figure S1a–c: *Ranunculus kuepferi*, Characteristics of diploid (2*x*) and tetraploid (4*x*) study plants; Figure S2a–d: Transect sites for the common garden experiment on *R. kuepferi* (Stubai Alps); Figure S3a–e: Details on the plot setup in the transect; Figure S4: *Ranunculus kuepferi*, natural 2*x* population (NAT), Western Alps, Piemont, Italy (Colle della Lombarda); Figure S5: *Ranunculus kuepferi*, natural 4*x* population (NAT), Eastern Alps, Tyrol, Austria, (Kaun Valley); Figure S6a,b: Snowfields and thawing lines at the natural sites of *R. kuepferi*. Figure S7a–g: Microscopic images of pollen grains and ovules of diploid and tetraploid *R. kuepferi* plants; Figure S8: Pollen grain size classes in R. kuepferi; Table S1: Ranunculus kuepferi. Provenances of diploid (2*x*) and tetraploid (4*x*) study plants; Table S2: Ranunculus kuepferi. Test parameters for reproductive development; Table S3: Reproductive mode of *R. kuepferi* in the transect; Table S4: Climatological and phenological dates during the growth period of *R. kuepferi.*

## Abbreviations

DAA: days after onset of anthesis; FCSS: flow cytometric seed screening; GP: Geographical Parthenogenesis; NAT: natural populations; P1800, P2300, P2600, P2800: plot sites in the transect at the respective elevation; P/O: pollen ovule ratio.
