# Supplementary material for: Reproductive Performance of the Alpine Plant Species Ranunculus kuepferi in a Climatic Elevation Gradient: Apomictic Tetraploids Do Not Show a General Fitness Advantage over Sexual Diploids
Source: Life (Basel). 2024 Sep 22;14(9):1202. doi: 10.3390/life14091202 (PMC11433044; doi:10.3390/life14091202)
Supplement: Supplementary file 1 [file life-14-01202-s001.zip › life-3158518-supplementary.pdf]

Supplementary Materials

Reproductive performance of the alpine plant species *Ranunculus kuepferi* in a climatic elevation gradient: apomictic tetraploids do not show a general fitness advantage over sexual diploids

Ursula Ladinig[1], Elvira Hörandl[2], Simone Klatt[2,3], Johanna Wagner*[1]

[1] Department of Botany, Functional Plant Biology, University of Innsbruck, Sternwartestrasse 15, A-6020 Innsbruck, Austria
[2] Department of Systematics, Biodiversity and Evolution of Plants, Albrecht-von-Haller Institute for Plant Sciences, University of Goettingen, Untere Karspuele 2, D-37073, Goettingen, Germany.
[3] Central administration, University of Goettingen, Humboldtallee 15, 37073, Goettingen, Germany

*Correspondence: johanna.wagner@uibk.ac.at

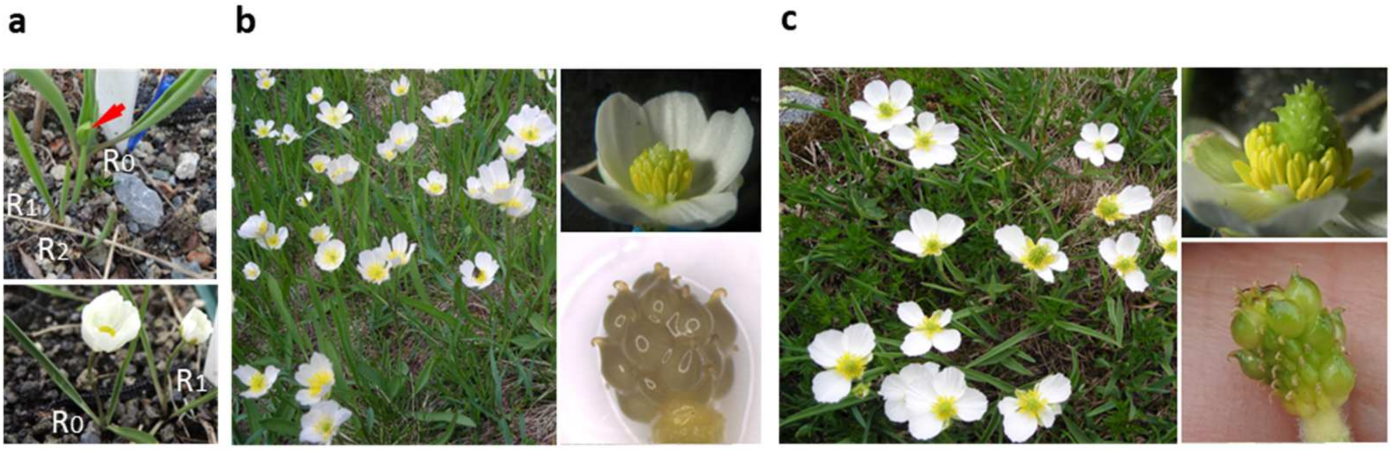

Figure S1. *Ranunculus kuepferi*. Characteristics of diploid (2*x*) and tetraploid (4*x*) study plants. (**a**) diploid individuals growing in plots; upper picture: main shoot (R_0) with a flower bud (red arrow) and two new vegetative lateral ramets (R_1, R_2); lower picture: main shoot and new lateral ramet with uniflorous inflorescence each. (**b**) diploid plants and (**c**) tetraploid plants in natural populations, and the typical appearance of a flower and an aggregate fruit consisting of a variable number of fruitlets (=achenes) whose development often failed in tetraploid plants.

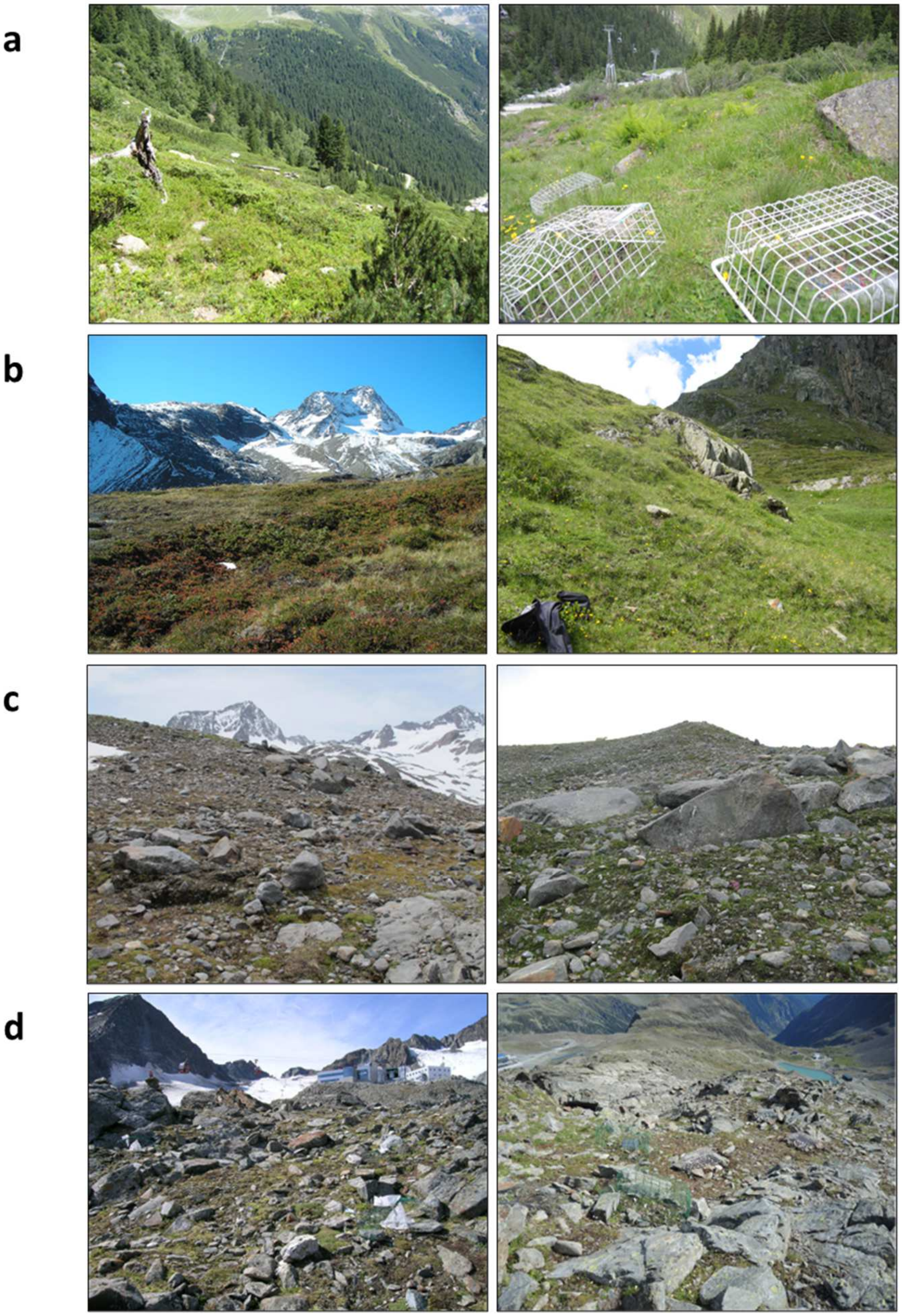

Figure S2a-d Transect sites for the common-garden experiment on *R. kuepferi*
Elevational transect, Stubai Alps, Eastern Central Alps, Austria
(**a**) Subalpine plot site P1800, 1770 m a.s.l., 47°00' N, 11°09’ E
(**b**) Lower alpine plot site P2300, 2290 m a.s.l., 46° 59' N, 11°08’ E, Austria
(**c**) Higher alpine plot site P2600, 2600 m a.s.l., 46° 59’46’’N, 11°07’29’’E
(**d**) Subnival plot site P2800, 2830 m a.s.l., 46° 59’18’’ N, 11°07’4’’E

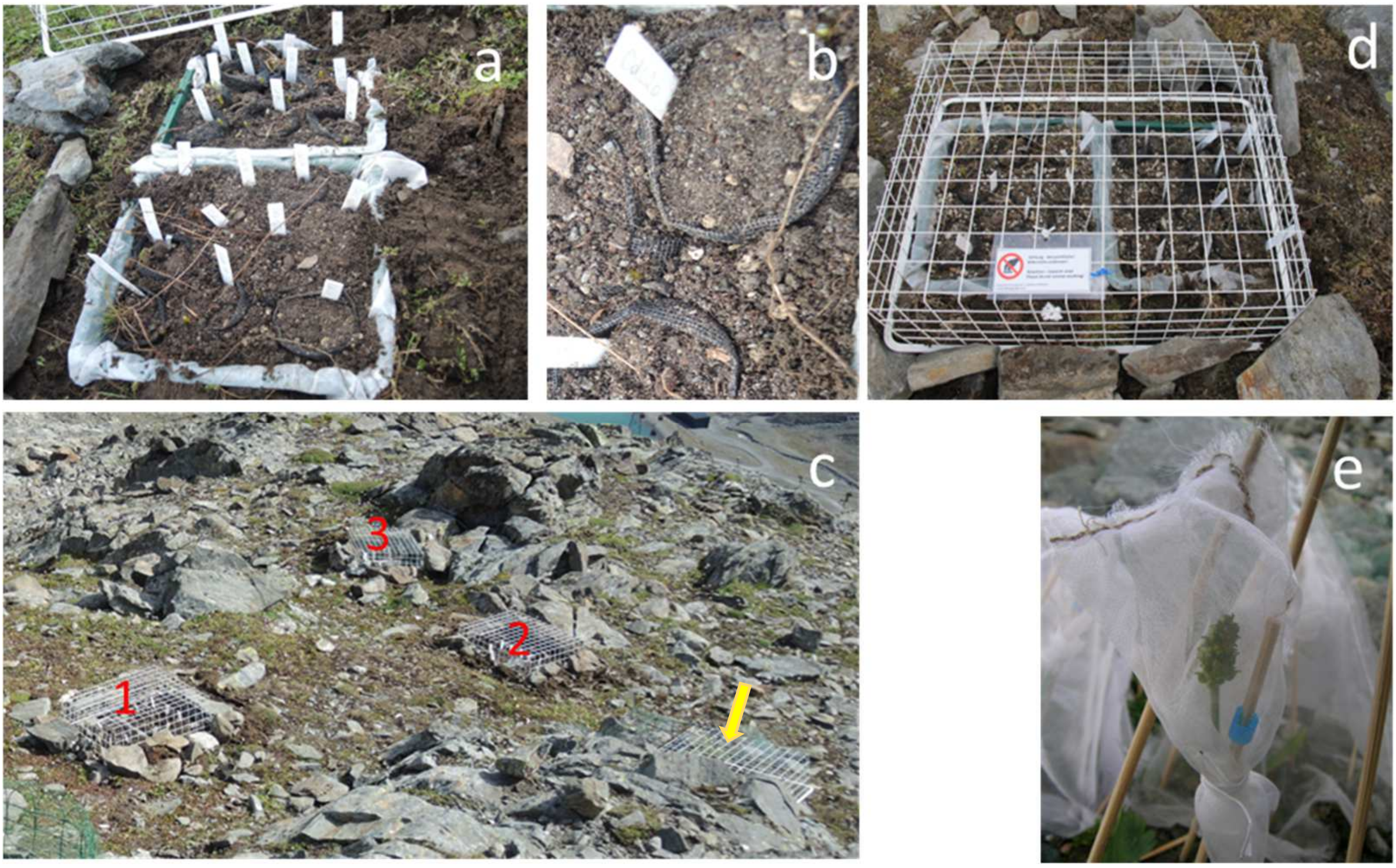

Figure S3 Details on the plot setup in the transect
(**a**) Single plot consisting of two plastic grid boxes containing 18 *R. kuepferi* individuals
(**b**) Individuals were delimited with a plastic mesh.
(**c**) Arrangement example of the 3 plots for tetraploids and a reserve plot (yellow arrow) at the P2800 site.
(**d**) Plot protected by a wire basket.
(**e**) Aggregate fruit bagged to avoid loss of achenes.

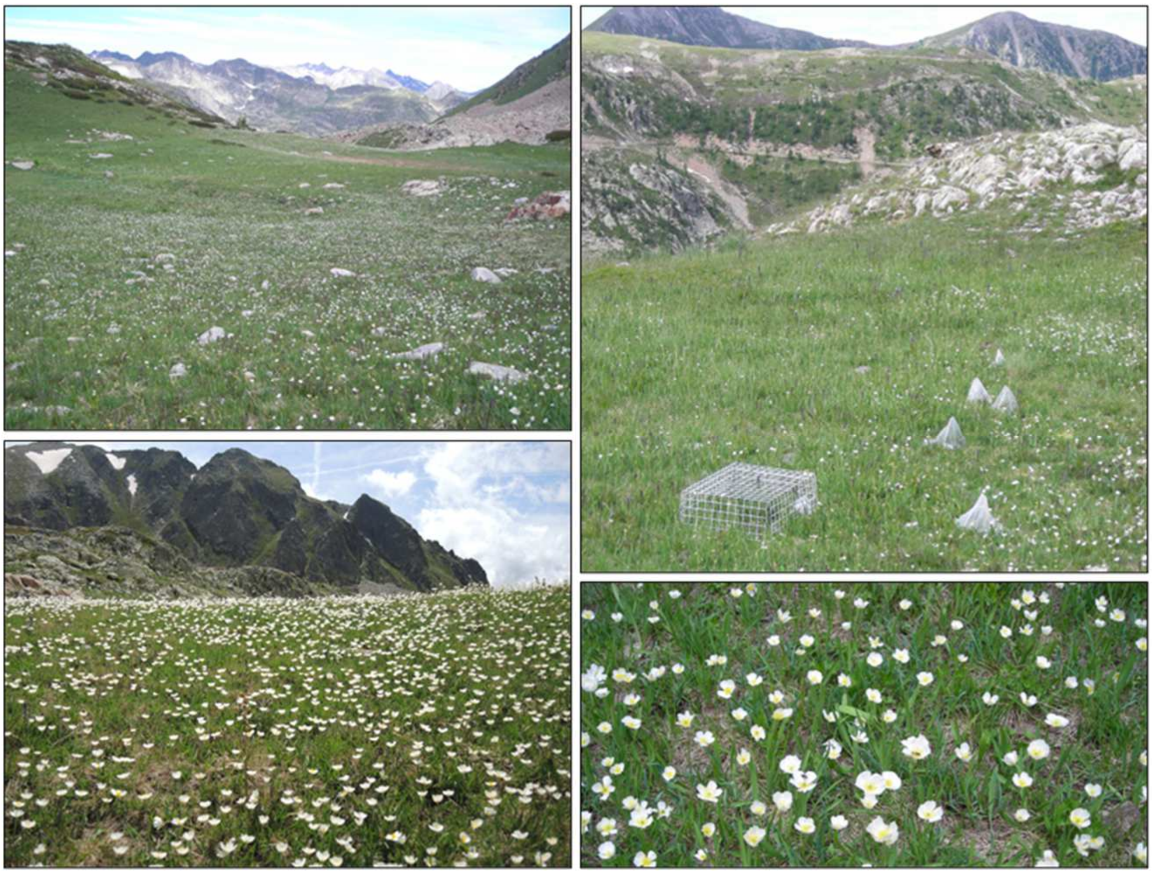

Fig. S4. ***Ranunculus kuepferi,*** **natural 2*x* population (NAT), Western Alps, Piemont, Italy**
Colle della Lombarda, 2260 - 2350 m a.s.l., 44°12′41′′N, 7°8′40′′E - 44°12′55′′N, 7°8′51′′E

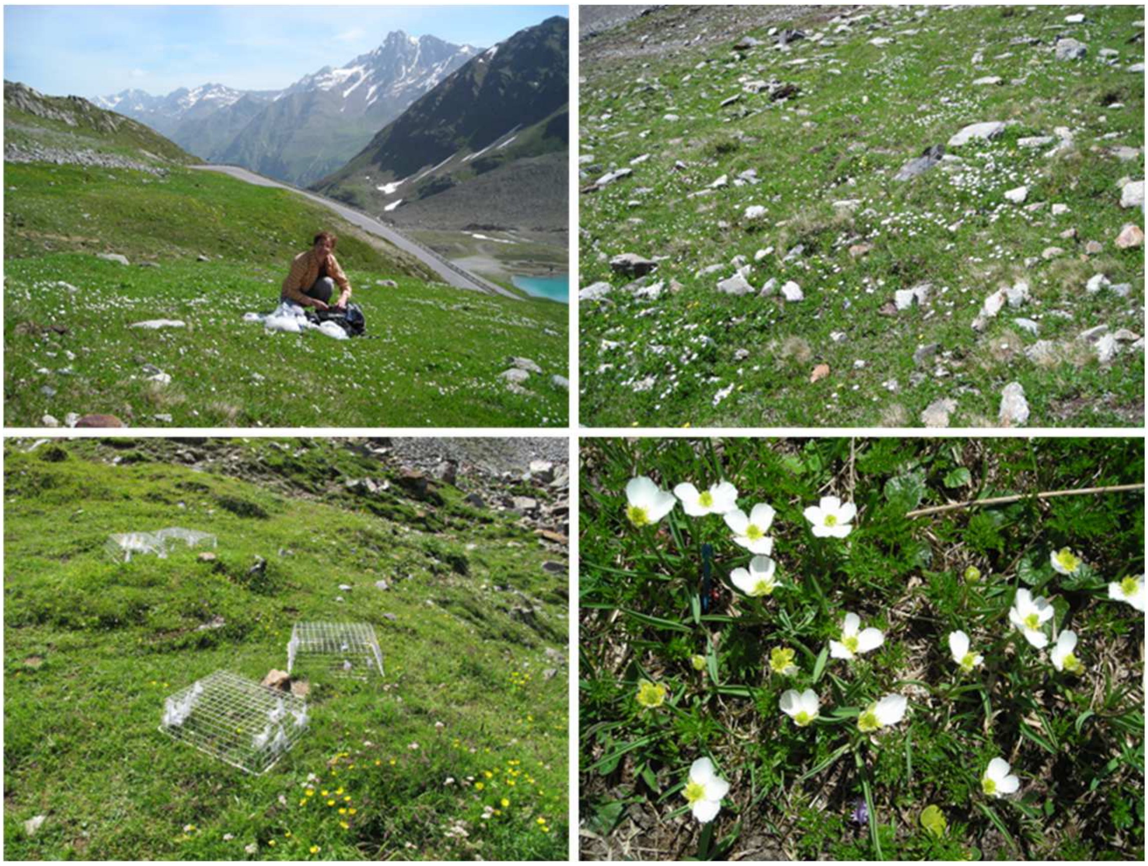

Fig. S5. ***Ranunculus kuepferi,*** **natural 4*x* population (NAT), Eastern Alps, Tyrol, Austria**
Kaun Valley, 2470-2580 m a.s.l., 46°52′18′′N, 10°42′41′′E - 46°52′15′′N, 10°42′32′′E

Figure S6a,b. Snow fields and thawing lines at the natural sites of *R. kuepferi*. To monitor the time course of phenophases, individuals were marked with coloured plastic pickers just as they emerged from the winter-snow at the edge of snowfields (red line)

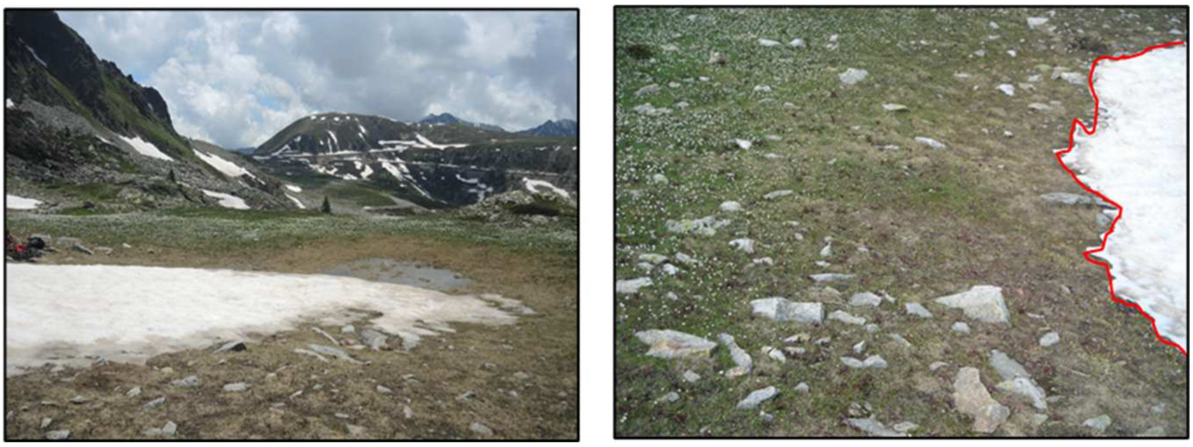

Figure S6a Colle della Lombarda, diploid sexual population

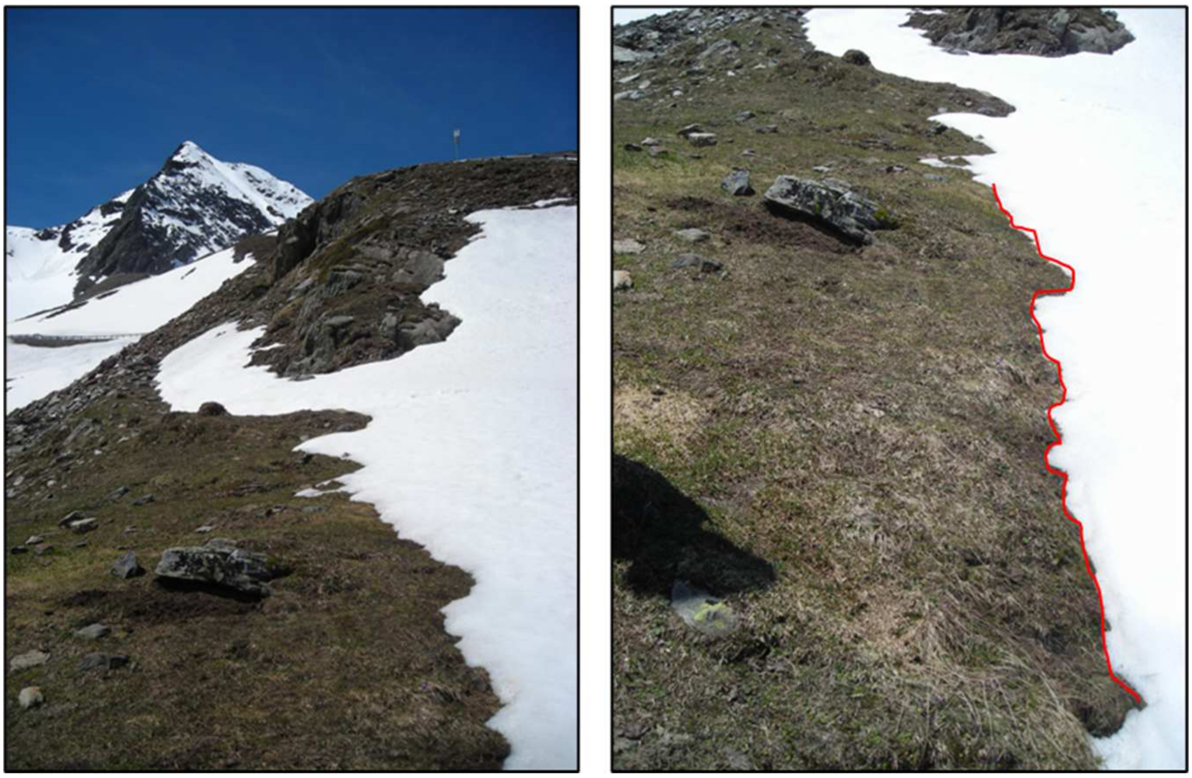

Figure S6b Kaun Valley, tetraploid apomictic population

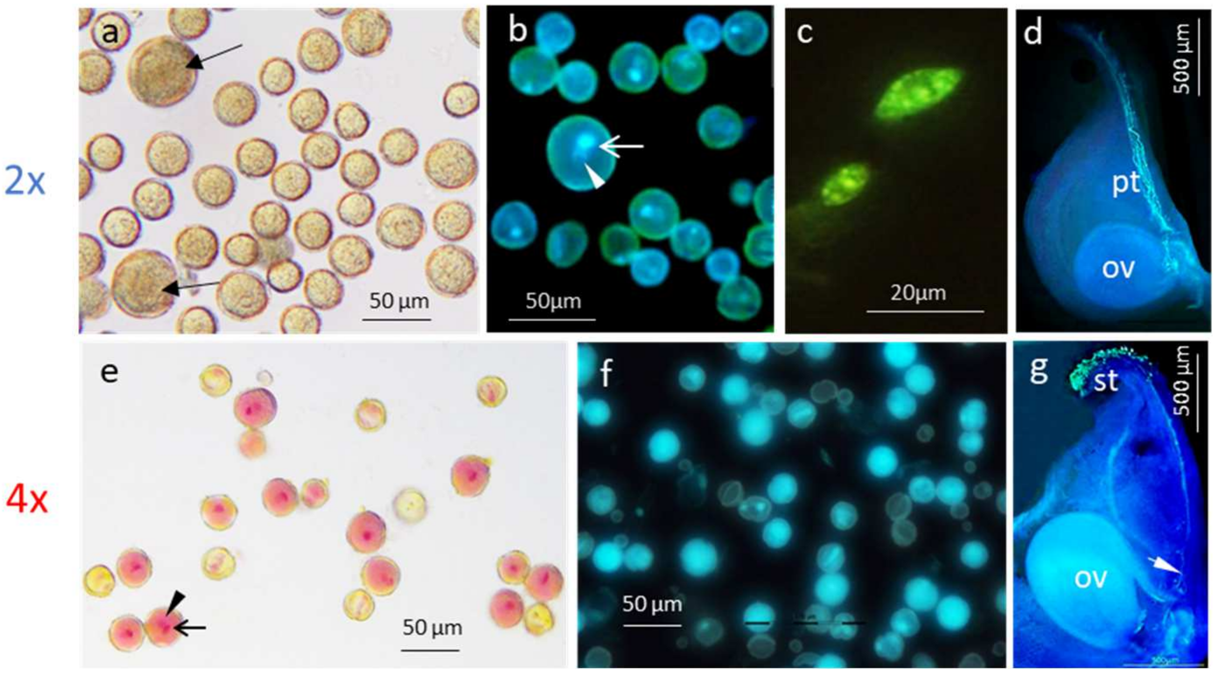

Figure S7. Microscopic images of pollen grains and ovules of (**a-d**) diploid (2*x*) and (**e-g**) tetraploid (4*x*) plants. (**a**) viable normal sized and giant pollen grains (arrows); (**b**) viable giant pollen grain among smaller ones with clearly visible generative (arrow) and vegetative nucleus (arrowhead); DAPI staining; (**c**) isolated generative nuclei of a normal sized (below) and a giant (above) pollen grain; (**d**) aniline blue stained carpel of a diploid flower with several pollen tubes (pt) that had grown down the transmitting tract, one of which most likely has fertilized the ovule (ov); low pollen viability in tetraploid plants visualized by (**e**) acetocarmin staining (arrow: generative nucleus, arrowhead: vegetative nucleus) and (**f**) DAPI staining; (**g**) carpel of a tetraploid flower; many pollen grains had germinated on the stigma (st) but only one pollen tube (arrow) has grown towards the ovule (ov).

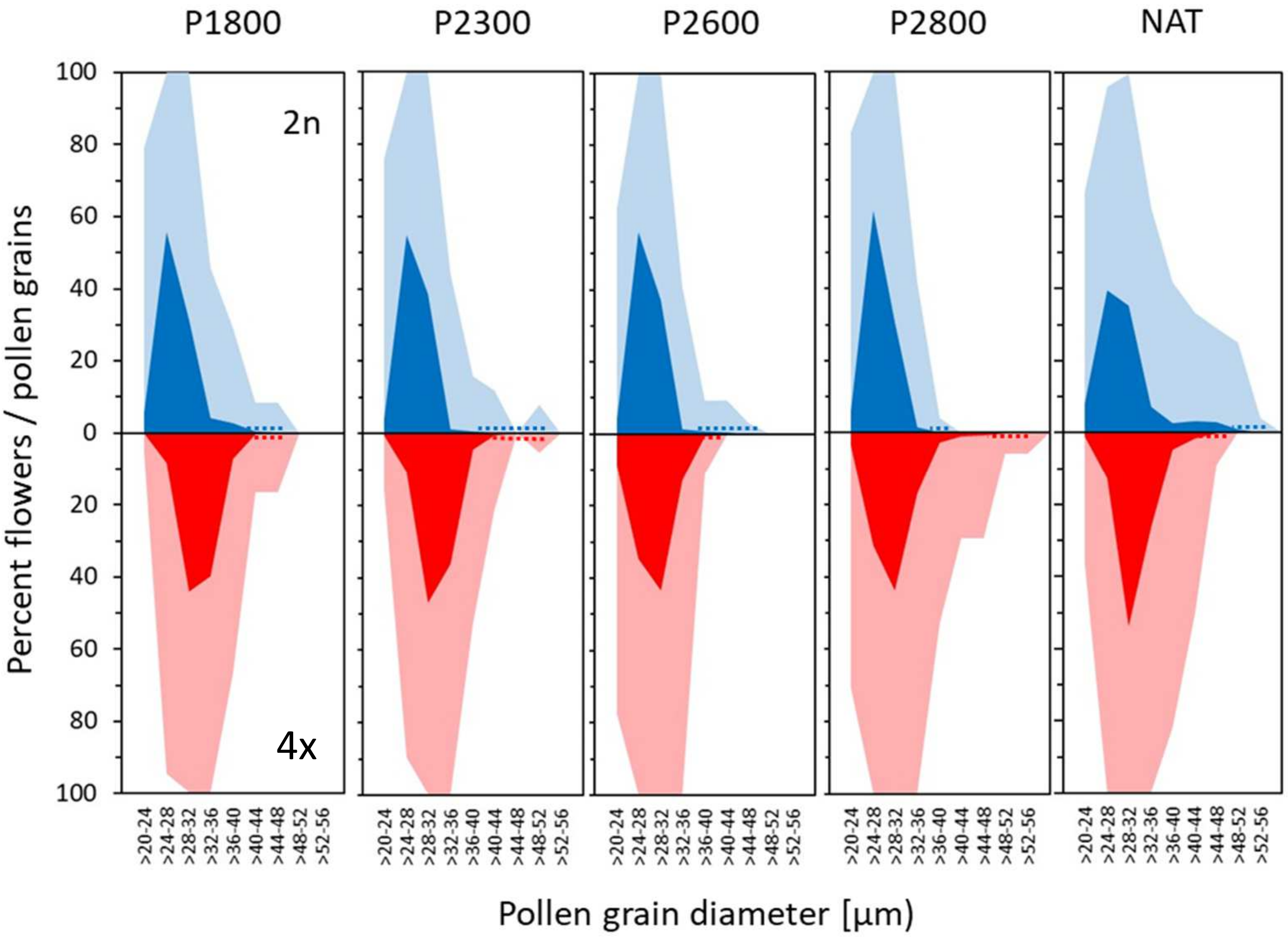

Figure S8. Pollen grain size classes in *R. kuepferi*. Percent pollen grains (dark colors) in the different size classes, and percent flowers (light colors) in which pollen grains in the respective size class occur. Broken line: percent pollen grains less than 1%. E.g. at the site P1800, all diploid flowers showed pollen grains in the size classes >24-32 µm, but only 8% of flowers contained also grains in the size classes >40-48; among all measured pollen grains at this site, most were found in the size class >24-28µm (56%), but less than 0.5 % in the size classes >40-48.

Table S1. *Ranunculus kuepferi*. Provenances of diploid (2*x*) and tetraploid (4*x*) study plants

Population no.	Cytotype[1)]	Country/ Region	Locality	Elevation m a.s.l.	Latitude N	Longitude E	Collector	No of individuals per plot / elevation
1	2x	Italy/ Piemont Western Alps	Colle della Lombarda	2260-2350	44°12’41” 44°12'55"	7°8’40” 7°08'51"	UL	13 / 39
2	2x	Italy/ Piemont Western Alps	Valle di Pesio	1700	44°14'40"	7°37'42"	UL	5 /15
3	4x	France/ Haute-Alpes Western Alps	Col de Vars	2112-2134	44°32'21“	6°42'06"	UL	3-4 / 9-10
4	4x	France/ Savoie Western Alps	Petit St Bernard	2197-2215	45°40’40’’	6°52’55”	UL	3 / 9
5	4x	France/ Savoie Western Alps	Col d‘Iseran	2250-2768	45°26'18"	7°0 ‘40“	UL	3-4 / 9-12
6	4x	Austria/ Tyrol Eastern Alps	Kaun Valley	2470-2580	46°52’18’’ 46°52’15’’	10°42’41’’ 10°42’32’’	UL	8-9 / 23-27

[1)] Cytotype and genetic structure of the populations are known from earlier investigations *[42]: Cosendai et al. 2013*, and *[51]: Cosendai and Hörandl 2010*. Study plants were collected in uniform diploid (2*x*) and tetraploid (4*x*) populations.

Table S2: *Ranunculus kuepferi.* Test parameters for the reproductive development, and the number of investigated units in the respective year(s) of investigation in both cytotypes. - not determined

	Figure			Transect plots				Natural
Test parameter	Table		Cytotype	P1800	P2300	P2600	P2800	sites
Phenology	F1, F3	study years		2015/16/17	2015/16/17	2015/16/17	2015/16/17	2014
		number of observed flowers	2x	41	39	33	45	76
			4x	48	52	21	42	70
Flower development	F1, F2	study years		2017-2018	2017-2018	2017-2018	2017-2018	2014-2015
		number of sampling dates /	2x	4 /5	3 / 6	7 / 7	7 / 9	8/ 13
		mean sampled individuals per date	4x	3 / 9	3 / 7	7 / 7	6 / 6	9 / 8
Dynamics of seed development	F6, F7	study year		2015	2015	2015	2015	2014
		number of labelled flowers	2x	32	17	21	22	150
			4x	29	25	15	24	150
Anther number	F8	study years		2015-2017	2015-2017	2015-2017	2015-2017	2014
		number of flowers	2x	47	51	59	62	54
			4x	61	42	40	47	70
Pollen grain number per anther and flower	F8	study year		2016	2016	2016	2016	2014
		number of flowers	2x	10	10	10	10	10
			4x	10	10	10	10	12
Pollen viability	F8	study years		2016-2017	2016-2017	2016-2017	2016-2017	2014
		number of flowers	2x	10+11	10+12	10+9	10+9	10+12
			4x	10+7	10+9	10	10+7	19+12
Intact pollen grains/flower	F8	study year		2016	2016	2016	2016	2014
		number of flowers	2x	10	10	10	10	10
			4x	10	10	10	10	12
Pollen grain size	F9, FS6	study year		2016	2016	2016	2016	2014
		number of measured pollen grains/	2x	1238/24	1296/25	1479/32	1232/24	1146/24
		in n flowers	4x	849/18	914/19	644/18	861/17	981/22
Ovule number/flower	F10	study years		2015-2017	2015-2017	2015-2017	2015-2017	2014
		number of flowers	2x	28	45	133	94	80
			4x	70	40	41	44	145
Ovule viability	F10	study years		2015-2017	2015-2017	2015-2017	2015-2017	2014
		number of flowers/analysed ovules	2x	20/114	21/109	28/202	20/113	38/170
			4x	26/231	21/97	27/198	22/108	54/252
Pollen/ovule ratio	F10	study year		2016	2016	2016	2016	2014
		number of flowers	2x	10	10	10	10	10
			4x	10	10	10	10	12
Stigma pollen load	T1	study year		2016	2016	2016	2016	2014
		number of flowers/analysed carpels	2x	11/63	8/40	13/101	8/60	27/164
			4x	11/75	8/59	15/102	14/98	23/307
Pollen germination	T1	study year		2016	2016	2016	2016	2014
		number of flowers/analysed carpels	2x	11/63	8/39	13/101	8/60	27/164
			4x	11/75	8/59	15/102	14/98	23/307
Pollen tube growth	T1	study year		2016	2016	2016	2016	-
		number of analyseable carpels from	2x	11	13	15	10	-
		30-60 prepared carpels per site and cytotype	4x	1	13	7	9	-
Seed set	T1	study years		2015/16/17	2015/16/17	2015/16/17	2015/16/17	2014
		number of flowers	2x	12/9/8	9/19/9	18/20/71	8/19/59	70
		examined	4x	16/19/36	15/15/12	6/6/30	9/11/26	95
Reproductive mode	TS3	study years		2014/2018	2014/2018	2014/2018	2014/2018	-
		number of seeds	2x	59	98	66	148	-
			4x	63	77	68	36	-

Table S3. Reproductive mode of *R. kuepferi* in the transect.
Summary of results of flow cytometric seed screening
From the 100 collected achenes per cytotype and site, a variable number of intact seeds could be obtained. On average, 5 seeds per individual were investigated.

Plot site	Ploidy	n individuals	n intact seeds	% apomictic seeds	% sexual seeds
P1800	2x	4	59	1.7	98.3
	4x	9	60	98.3	1.7
P2300	2x	14	98	0.0	100.0
	4x	11	77	96.1	3.9
P2600	2x	17	70	1.4	98.6
	4x	15	66	92.4	7.6
P2800	2x	41	162	0.0	100.0
	4x	13	50	94.0	6.0
Total	2x	76	389		
	4x	48	253		
Average/individual	2x		5.1		
	4x		5.3		

Table S4. Climatological and phenological dates at the natural sites, and in the plots along the elevational gradient during the growth period of *R. kuepferi* (snowmelt until seed maturity) in two years (Kaun Valley, KV-4x NAT site) and three years (transect and Colle della Lombarda, CdL-2x NAT site) of investigation.

A<br>Site	B<br>Mean snowmelt date	C<br>Mean temperature [°C±SD]	D<br>Days with snow cover	E<br>Period between snowmelt and seed maturity [d±SD]
Transect sites				
P1800-2x	8.-12. May	11.7±2.2 A	0-8	54.3±4.6
P1800-4x	5.-11. May	12.3±1.3 a	0-8	61.3±1.2
P2300-2x	15.-20. May	11.9±1.9 A	0-11	55.0±6.2
P2300-4x	15.-19. May	11.5±1.0 a	0-11	57.7±3.8
P2600-2x	9.-19. June	11.2±0.6 AB	0-3	56.3±13.1
P2600-4x	16.-30. June	11.9±3.2 a	0-3	54.7±14.2
P2800-2x	12.-24. June	10.0±1.0 B	0-4	70.7±6.7
P2800-4x	12.-21. June	9.6±0.9 b	0-4	78.7±5.1
Natural sites (NAT)				
CdL -2x	29. May-18. June	13.4±1.2 C	0	46
KV – 4x	5.-11. June	9.9±1.0 b	0	53

A: CdL Colle della Lombarda; K Kaun Valley
B: Mean snowmelt date in the investigation years
C: Mean daily mean temperature during the growth period of *R. kuepferi* (snowmelt until seed maturity)
D: Days with snow cover during the growth period of *R. kuepferi* in the years of investigation
E: Total period between snow melt and seed maturity

SD standard deviation of the annual means.
Mean temperature: Significant differences among sites are indicated by upper-case letters (2*x* plants) and lower-case letters (4*x* plants); (one-way ANOVA, Games-Howell post hoc comparison).
Differences between cytotypes at the same elevation along the transect are not significant, however highly significant ($P < 0.001$) at the natural sites (t-test).
